# Single-cell transcriptome analysis reveals the immune heterogeneity and the repopulation of microglia by Hif1α in mice after spinal cord injury

**DOI:** 10.1038/s41419-022-04864-z

**Published:** 2022-05-03

**Authors:** Jingyu Wang, Lintao Xu, Weiwei Lin, Yin Yao, Heyangzi Li, Gerong Shen, Xi Cao, Ning He, Jun Chen, Jue Hu, Mingzhi Zheng, Xinghui Song, Yuemin Ding, Yueliang Shen, Jinjie Zhong, Lin-lin Wang, Ying-ying Chen, Yongjian Zhu

**Affiliations:** 1 Department of Neurosurgery, Second Affiliated Hospital of Zhejiang University School of Medicine, Key Laboratory of Precise Treatment and Clinical Translational Research of Neurological Diseases, Hangzhou, China; 2 Clinical Research Center for Neurological Diseases of Zhejiang Province, Hangzhou, China; 3 Department of Neurointensive Care Unit, Second Affiliated Hospital of Zhejiang University School of Medicine, Hangzhou, China; 4 Department of Basic Medicine Sciences, Zhejiang University School of Medicine, Hangzhou, China; 5 School of Basic Medical Sciences & Forensic Medicine of Hangzhou Medical College, Hangzhou, China; 6 Core Facilities, Zhejiang University School of Medicine, Hangzhou, China; 7 School of Medicine, Zhejiang University City College, Hangzhou, China; 8 Department of Basic Medicine Sciences, and Department of Obstetrics of the Second Affiliated Hospital, Zhejiang University School of Medicine, Hangzhou, China; 9 Department of Basic Medicine Sciences, and Department of Orthopaedics of Sir Run Run Shaw Hospital, Zhejiang University School of Medicine, Hangzhou, China

**Keywords:** Microglia, Spinal cord injury

## Abstract

Neuroinflammation is regarded as a vital pathological process in spinal cord injury (SCI), which removes damaged tissue, secretes cytokines, and facilitates regeneration. Repopulation of microglia has been shown to favor recovery from SCI. However, the origin and regulatory factors of microglia repopulation after SCI remain unknown. Here, we used single-cell RNA sequencing to portray the dynamic transcriptional landscape of immune cells during the early and late phases of SCI in mice. B cells and migDCs, located in the meninges under physiological conditions, are involved in immune surveillance. Microglia quickly reduced, and peripheral myeloid cells infiltrated three days-post-injury (dpi). At 14 dpi, microglia repopulated, myeloid cells were reduced, and lymphocytes infiltrated. Importantly, genetic lineage tracing of nestin^+^ and Cx3cr1^+^ cells in vivo showed that the repopulation of microglia was derived from residual microglia after SCI. We found that residual microglia regress to a developmental growth state in the early stages after SCI. Hif1α promotes microglial proliferation. Conditional ablation of Hif1α in microglia causes larger lesion sizes, fewer axon fibers, and impaired functional recovery in the late stages after SCI. Our results mapped the immune heterogeneity in SCI and raised the possibility that targeting Hif1α may help in axon regeneration and functional recovery after SCI.

## Introduction

The central nervous system (CNS) is considered an area with immune privilege [1]. Spinal cord injury (SCI) leads to dramatic changes in the immune landscape [2]. Resident microglia quickly reduce and transfer to a reactive state, followed by the infiltration of peripheral immune cells [3]. Peripheral neutrophils are initially recruited to the lesion core, followed by monocytes, macrophages, and DCs [4]. Lymphocyte infiltration occurs during chronic inflammation [5]. Full immune diversity in SCI remains poorly characterized. To explore the immune landscape in uninjured and injured spinal cords, we sorted CD45^+^ immune cells and conducted single-cell RNA-sequencing (scRNA-seq).

Microglia, a type of tissue-resident macrophage with dense ramifications, are the main immune cells in the CNS that respond to injury rapidly [6]. After injury, microglia can be roughly divided into ‘quiescent’ and ‘reactive’ subsets. The identification of reactive microglia depends on morphological features [7]. Previous studies have characterized different subtypes of microglia during physiology and in several diseases, including neurodegeneration, acute injury, aging, Alzheimer’s disease, and multiple sclerosis [8–12]. Over-reactive microglia that secrete excess inflammatory cytokines induce neurotoxic reactive astrocytes and cause secondary injury [13]. Instead, microglia can phagocytize myelin debris and dead cells, produce protective scars for wound compaction, and promote re-myelination [14]. Importantly, impairment of locomotor recovery can be caused by the removal of microglia after SCI, whereas microglial repopulation benefits functional recovery [15]. Additionally, reborn microglia possess neuroprotection through the IL-6 pathway after brain injury [16]. The beneficial effects of reborn microglia after CNS injury may outweigh their harmful effects. Thus, promoting microglial repopulation may be a potential therapy for SCI.

At first, it is imperative to identify the regulatory factors for microglial repopulation. SCI can result in extensive hypoxia in the lesion due to the mechanical destruction of blood vessels, increased infiltration of immune cells, proliferation of glial cells, and constriction of capillaries at pericytes [17, 18]. Hypoxia-inducible factor 1α (Hif1α) is expressed by cells in response to hypoxia. Degradation of Hif1α is mediated by von Hippel-Lindau (VHL) E3 ubiquitin in normoxia, which is relieved in hypoxia and then the accumulation of Hif1α is present in cells [19]. The Hif1α pathway promotes the proliferation of granule neuron precursors in the postnatal phase. However, degradation of Hif1α controls neuron differentiation [20]. Microglial proliferation and the Hif1α pathway occur simultaneously in the early phase of SCI in our scRNA-seq data. Ingenuity Pathway Analysis (IPA) of differentially expressed genes (DEGs) in proliferative microglia revealed that the Hif1α-Zeb1 axis might be a critical upstream regulatory factor. Thus, we next explored the role of Hif1α in the repopulation of microglia after SCI and the effect of Hif1α in microglia on functional recovery was explored.

## Materials and methods

### Materials

The details of the materials are shown in Supplementary Table S1.

### Animals

All experiments involving animals were conducted in accordance with the guidelines of the Institutional Animal Care and Use Committee and approved by the Ethics Committee of Zhejiang University (No. ZJU20210190). All mice were maintained at the Laboratory Animal Center of Zhejiang University under specific pathogen-free conditions, and housed with three to five mice per cage under 12-h light/dark cycle with ad libitum access to sterile water and food. All mice were maintained on a C57/BL6 background. All mice were divided into certain groups randomly using a random number method.

Hif1α^flox^ mice were established by Shanghai Model Organisms Center, Inc. (SMOC). Ai9, Nestin^CreERT2^, and CX3CR1^CreERT2^ mice were obtained from the Jackson Laboratory [21, 22]. CX3CR1^CreERT2^:: Ai9 and Nestin^CreERT2^:: Ai9 mice were used for lineage tracing, as previously described [23]. CX3CR1^CreERT2^:: Ai9 mice were intraperitoneally injected with tamoxifen (150 mg/kg) once daily for four days at three to four weeks old to induce microglia to constantly express tdT. Nestin^CreERT2^:: R26 ^tdTomato^ mice were treated with tamoxifen to induce tdT expression in Nestin^+^ cells and left to rest for two weeks.

### SCI induction

Three-month-old mice were deeply anesthetized with isoflurane inhalation. The SCI mouse model was established via Allen’s method, as described previously [24, 25]. Briefly, mice underwent laminectomy at thoracic spinal segment T10. Then, NYC-II struck instrument was then used to produce a moderate contusion on the exposed spinal cord under a force of 5 g × 15 mm. The same surgical operation without contusion was performed in sham mice. SCI mice underwent bladder massage to aid urination twice daily across the experiments. All mice were subcutaneously injected with 1 mg/kg flunixin meglumine, 5 mg/kg ceftiofur sodium, and 0.5 ml of saline once daily for five days after surgery.

### Spinal cord single-cell dissociation

Mice were anaesthetized and transcardially perfused with ice-cold saline. Injured or normal spinal cord segments (8 mm) centered on the lesion site were immediately extracted and maintained in an ice-cold Hibernate-A medium (Gibco). Single-cell suspensions were obtained by using a Neural Tissue Dissociation Kit (P) (Miltenyi Biotec) according to the manufacturer’s protocol. Briefly, the segments were cut into small pieces and then incubated in 50 μL Enzyme P diluted in 1.95 mL Buffer X for 30 min at 37 °C with gentle shaking (100 r.p.m). Enzyme A (10 μL) diluted in 20 μL of Buffer X was next added to the digestive system. Following incubation for 10 min, single-cell suspensions were generated by pipette blowing. The suspensions were then strained using a cell strainer (70 μm) and centrifuged. Cell pellets were resuspended in 33% Percoll (GE Healthcare) layered on 70% Percoll and centrifuged for 15 min at 1800 r.p.m. Cells were obtained from the 30–70% interface and washed with Hank’s Balanced Salt Solution twice. All steps were conducted at 4 °C except digestion.

For ActD based dissociation, ActD (Sigma-Aldrich) was added across all steps during the dissociation at the final concentration of 45 μM at 37 °C for digestion or 3 μM at 4 °C for trituration and cell sorting [26].

### Cell sorting

For single-cell RNA sequencing (scRNA-seq), mice (*n* = 30, 15 males, 15 females) were randomly divided into three groups (control; three and 14 dpi). After experimental processing, single-cell suspensions were acquired as described above, washed twice in fluorescence-activated cell sorting (FACS) buffer (PBS supplemented with 0.2% B27 and 0.1% N2 nutrient), and incubated with an anti-CD16/32 antibody (Fc blocker, BD Biosciences) for 5 min at 4 °C, Cell pellets were then stained with an anti-CD45 antibody (BD Bioscience) for 20 min at 4 °C and washed twice. For cell sorting, live cells were stained with DAPI (BD Biosciences), and CD45 positive cells were purified using Beckman Moflo Astrios EQ.

### Single-cell RNA sequencing

ScRNA-seq was conducted using the 10× Chromium Single Cell Platform (Single Cell 3′ library and Gel Bead Kit v.3) according to the manufacturer’s protocol [27]. Library quantification was performed with Qubit and the library pool was sequenced on an Illumina NovaSeq 6000 sequencing platform using 150 base pair paired-end reads.

Quality control metrics were calculated using the R package Seurat v.3.1. Genes were expressed in fewer than ten cells. We filtered cells with more than 10% mitochondrial genes or fewer than 600 distinct genes. Cells expressing both myeloid and lymphoid markers (co-expressed Itgam and CD3e, Itgam and CD19, or Itgam and Klrb1c) were removed. R package DoubletFinder was used to detect and remove doublets [28].

Seurat’s LogNormalize method was used to normalize and scale the data for sequencing depth (scale.factor = 10,000). Highly variable genes were detected using Seurat’s FindVariableGenes function: x.low.cutoff = 0.0125, x.high.cutoff = 3, y.cutoff = 0.5. For the integration of different samples, Seurat’s integration workflow was used to bath-correct the expression matrices (anchor_n_cutoff < −2000; k.filter_cutoff < −200; anchor_pca_n_cutoff < −50) [29]. Cells were visualized by t-distributed stochastic neighbor embedding (t-SNE) and clustered using the FindClusters method (resolution = 0.6). FindAllMarkers function was used to identify differentially expressed genes (DEGs). DEGs with p_val_adj dj l_ion waavg_logFC vg_logFC were defined as significant DEGs.

GO analysis (biological process) was performed using Metascape ((http://metascape.org) [30], which was visualized using a bioinformatics platform (http://www.bioinformatics.com.cn/).

### Flow cytometry

Single-cell suspensions were prepared as previously described. Cells were washed twice FACS buffer followed by incubation with an anti-mouse CD16/32 antibody at 4 °C for 5 min. Cell pellets were then stained with antibodies (listed in Supplementary Table S1) for 20 min at 4 °C in the dark. Subsequently, the cells were washed twice and resuspended in FACS buffer. DAPI was used to exclude dead cells. Samples were analyzed using a CytoFlex LX Flow Cytometer (Beckman).

According to our scRNA-seq data and previous studies [1, 31, 32], we defined B cells as CD45^high^ CD19^+^ cells; NK cells as CD45^high^ CD19^−^ NK1.1^+^ CD3e^−^ cells; NK T cells as CD45^high^ CD19^−^ NK1.1^+^ CD3e^+^ cells; αβ T cells as CD45^high^ CD19^−^ NK1.1^−^ CD3e^+^ TCR β^+^ cells; γδ T cells as CD45^high^ CD19^−^ NK1.1^−^ CD3e^+^ TCR β^−^ cells; neutrophils 1 as CD45^+^ CD11b^+^ Ly6g^−^ CxCr2^+^ cells; neutrophils 2 as CD45^+^ CD11b^+^ Ly6g^+^ CxCr2^+^ MHC II^+^ cells; neutrophils 3 as CD45^+^ CD11b^+^ Ly6g^+^ CxCr2^+^ MHC II^−^ cells; neutrophils 4 as CD45^+^ CD11b^+^ Ly6g^+^ CxCr2^−^ cells; microglia as CD45^+^ CD11b^+^ Csf1r^+^ Cx3Cr1^+^ cells; homeostatic macrophage as CD45^+^ CD11b^+^ Csf1r^+^ Cx3Cr1^−^ CD63^−^ CD206^+^ cells; injury associated macrophage 1 as CD45^+^ CD11b^+^ Csf1r^+^ Cx3Cr1^−^ CD63^+^ cells; injury associated macrophage 2 as CD45^+^ CD11b^+^ Csf1r^+^ Cx3Cr1^−^ CD63^+^ CD206^+^ cells.

### Immunohistochemistry

Mice were anesthetized and perfused with ice-cold PBS and 4% paraformaldehyde. Healthy or injured spinal cord segments were dissected, fixed overnight at 4 °C, and dehydrated for two days. The spinal cord segments were embedded in Tissue-Tek O.C.T Compound (Thermo Fisher). Sagittal or transverse sections were obtained using a freezing microtome (Thermo Fisher, NX50). The sections were blocked with PBS containing 10% donkey serum, 1% BSA, and 0.25% Triton X-100 for 1 h at room temperature. Subsequently, sections were incubated with primary antibodies at 4°C overnight. The sections were incubated with secondary antibodies (1:500) at room temperature. DAPI (1:100) solution was used for nuclear staining. Sections were imaged using an Olympus *FV3000* confocal microscope.

The following primary antibodies were used: anti-CD19 (Rat, 1:200; Invitrogen); GFAP (Chicken, 1:500; Abcam); Nkp (goat, 1:200; R and D Systems); TCR β (Hamster, PE conjugated, 1:100; BD Biosciences); IL17a (Rat, Alexa Fluor 488 conjugated, 1:100; Invitrogen); Ccr7 (Rabbit, 1:500; Abcam); Tmem119 (Rabbit, 1:500; Abcam); RFP (Rabbit, 1:100; Rockland); RFP (Chicken, 1:100; Rockland); Ki67 (Rabbit, 1:200; Invitrogen); c-Myc (mouse, 1:200; Novus); Spp1 (goat, 1:200; R and D Systems); Nestin (Chicken, 1:200; Novus); IBA1 (Rabbit, 1:500; Abcam); NF-H (Chicken, 1:500; Abcam); CD31 (goat, 1:200; R and D Systems).

The following secondary antibodies from Jackson ImmunoResearch were used: Alexa Fluor 488 conjugated donkey anti-rat; Alexa Fluor 594 conjugated donkey anti-chicken; Alexa Fluor 488 conjugated donkey anti-chicken; Alexa Fluor 488 conjugated donkey anti-mouse; Alexa Fluor 488 conjugated donkey anti-rabbit; Alexa Fluor 488 conjugated donkey anti-goat.

### Detection of hypoxia in the spinal cord after SCI

Hypoxia in the tissue was detected using a Hypoxyprobe kit (Hypoxyprobe, Inc) according to the manufacturer’s protocol. Briefly, mice were intraperitoneally injected with the Hypoxyprobe™-1 (60 mg/kg). After 1 h, spinal cord sections were collected as above described, and next incubated with an anti-pimonidazole antibody (1:500) overnight at 4 °C. After washing three times with PBS, the sections were incubated with a FITC-conjugated IgG1 mouse monoclonal antibody (1:100) for 2 h at room temperature. FITC^+^ cells had an oxygen concentration of <14 μM. Sections were imaged using an Olympus *FV3000* confocal microscope.

### qRT-PCR

Total RNA was extracted from CD45^+^ cells sorted from spinal cords using TRIzol (Thermo Fisher) with standard or ActD-based dissociation methods. cDNA synthesis was performed using the PrimScript RT Reagent Kit with gDNA Eraser (Takara) according to the manufacturer’s protocol. Quantitative RT–PCR was performed using 100 ng cDNA and TB Green Premix Ex Taq II (Takara) on LightCycler 480 II system (Roche) with a cycling program of 95 °C for 30 s, followed by 40 cycles of 5 s at 95 °C, and 1 min at 60 °C. A melting curve of the amplified products was obtained using the above program. Data was collected and analyzed using the LightCycler 480 Software (1.5.0).

### Basso mouse scale (BMS) scoring

BMS scoring was performed to evaluate hind limb locomotor activity in an open field for 5 min as previously described [33]. Mice underwent bladder evacuation and were adapted to the testing environment for 1 h before testing. The initial score was similar for all mice. The observation began at the same time on each test day (13:00), and two observers were blinded to the groups, treatments, and genotypes. Mice with a difference of more than 2 score points between the left and right hindlimbs were excluded.

### Rotarod testing

Coordination and gross motor capability were assessed by rotarod testing [34, 35]. The mice were habituated to the test room for 1 h before testing. An accelerating rotarod was used to measure gross motor capability and coordination by accelerating the rod from 0 to 40 r.p.m. Each mouse was subjected to one practice trial, followed by two test trials, with an interval of 20 min between trials. The latency to fall was recorded and averaged from two test trials per mouse.

### Von Frey filament testing

The von Frey filament test was performed to evaluate hind limb tactile sensory recovery [35]. Mice were acclimated to a metal-mesh-bottom cylinder for 30 min. The lateral area of left/right hind paws was stabilized with a full set of von Frey filaments ranging from 0.004 *g* to 8 *g*. The stimulus was repeated five times. Hind paw licking or withdrawal represents three out of five stimuli marked as the von Frey threshold.

### Statistical analysis

The data were analyzed by GraphPad Prim (8.0). Although no statistical methods were used to pre-determin sample sizes, sample sizes in our study are similar to those in other publications. Two groups of data were analyzed by Student’s t tests. One-way ANOVA with Tukey’s multiple comparisons posttest or two-way ANOVA-RM with Bonferroni’s post hoc correction were used when compared multiply groups. The data are presented as the mean ± standard error of the mean (SEM) and statistical significance was set at *p* < 0.05.

## Results

### Isolation of immune cells using actinomycin D to minimize the artificial activation of IEGs

To recognize various immune cell subtypes in the lesion after SCI, scRNA-seq was performed using a 10× platform (Supplementary Fig. S1A). However, induction of transcriptional perturbations, especially IEGs, changes cell states [36, 37]. Adding ActD during the preparation of single-cell suspensions could minimize IEGs [26]. Compared with other cell types in the amygdala, IEGs were strongly upregulated in microglia, which is an important immune cell type in our study. We obtained a total of 4467 CD45^+^ immune cell transcriptomes from sham groups undergoing the standard protocol (2870 cells) or ActD-based dissociation protocol (1597 cells). Six distinct clusters were represented using t-distributed stochastic neighbourembedding (t-SNE), including microglia, macrophage, monocytes, and monocyte-derived cells, neutrophils, and B cells (Supplementary Fig. S1B). These cell types were defined based on key signature markers [1, 10, 38].

There was little overlap in the microglia between these two samples on t-SNE (Supplementary Fig. S1C). Analysis of DEGs revealed elevated expression of many IEGs as previously described, when cells were dissociated using a standard procedure (Supplementary Fig. S1D) [26]. Real-time qPCR further verified that ActD significantly reduced the expression of IEGs (Supplementary Fig. S1E). Cell clusters that were dissociated using standard methods highly expressed IEGs, particularly microglia (Supplementary Fig. S1F). Taken together, these results revealed that the addition of ActD during single-cell preparation minimized the artificial induction of IEGs, especially in microglia.

### Unbiased scRNA-seq reveals immune cell heterogeneity in the uninjured and injured spinal cords

**Fig. 1:**
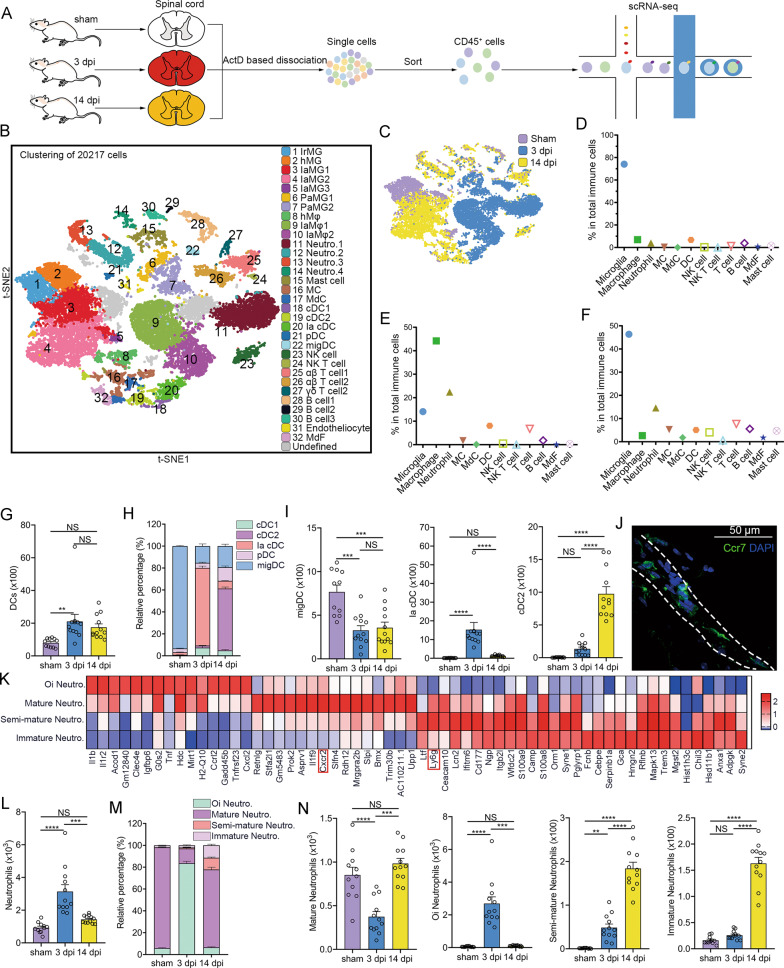
DCs and neutrophil heterogeneity after SCI. **A** Experimental design for scRNA-seq of CD45^+^ cells dissociated from healthy and injured spinal cords. **B** A t-SNE plot of 20,217 CD45^+^ cells isolated from the spinal cord after SCI at 0, 3, and 14 dpi. IaMG, injury-associated microglia; PaMG, proliferation-associated microglia; hMφ, homeostatic macrophage; IaMφ, injury-associated macrophage; cDC, classical dendritic cell; pDC, plasmacytoid dendritic cell; migDC, migratory dendritic cell; MdF, monocyte-derived fibrocyte. **C** A t-SNE plot with the colors representing cells from different samples. **D**–**F** The proportion of various immune cell types at sham, 3 dpi, and 14 dpi, respectively. **G** Flow cytometric analyses show the absolute number of DCs after SCI per mouse. One-way ANOVA with Tukey’s multiple comparisons posttest, *n* = 11 mice in the sham group, *n* = 12 mice in the 3 dpi and 14 dpi groups, **P = 0.0089. **H** Flow cytometric analyses show the proportion of different DC subsets in total DCs per mouse. **I** Flow cytometric analyses show the change of different DC subsets per mouse after SCI, respectively. One-way ANOVA-RM with Bonferroni’s post hoc correction. ***P = 0.0001 (sham vs. 3 dpi); ***P = 0.0003 (sham vs. 14 dpi); ****p < 0.0001. **J** An IHC image of a transverse section in the sham group shows the location of migDC. They are mainly in the dura mater. A dashed area outlines meninges. Images indicate similar results from three independent mice. Scale bar: 50 μM. **K** A heatmap shows the normalized expression for markers in neutrophil subsets. **L** Flow cytometric analyses show the absolute number of neutrophils per mouse after SCI. One-way ANOVA with Tukey’s multiple comparisons posttest, *n* = 11 mice in the sham group, *n* = 12 mice in the 3 dpi and 14 dpi groups, ***P = 0.0002, ****P < 0.0001. **M** Flow cytometric analyses show the proportion of different neutrophil subsets in total neutrophils per mouse. **N** Flow cytometric analyses show the change of different neutrophil subsets per mouse after SCI, respectively. One-way ANOVA-RM with Bonferroni’s post hoc correction. ***P = 0.0001; **P = 0.0054; ****P < 0.0001.

To assess immune cell heterogeneity at the injured sites, Act-seq was performed on CD45^+^ cells sorted from the uninjured and injured spinal cord segments at 3 and 14 dpi (Fig. 1A). We obtained 20,217 immune cells (2,131 cells in the sham group, 8,363 cells at three dpi, and 9,723 cells at 14 dpi). Unbiased clustering analysis identified 32 distinct cell clusters according to specific markers, consisting of seven clusters of microglia, three clusters of macrophage, four clusters of neutrophils, mast cells, monocytes, monocyte-derived cells, five clusters of dendritic cells, NK cells, NK T cells, three clusters of T cells, three clusters of B cells, endotheliocyte, and monocyte-derived fibroblast (Fig. 1B, S3A). The proportion and cell state of immune cells markedly changed in SCI (Fig. 1C and Supplementary Fig. S3B, C). IEGs were mostly expressed in clusters (Clusters 6, 7, 9, 10, and 11) mainly derived from the 3 dpi group (Supplementary Fig. S3C, D).

Microglia are the main immune cells (approximately 78%) in the healthy spinal cord, followed by macrophages (approximately 7%) (Fig. 1D). SCI resulted in a microglial reduction at 3 dpi, with infiltration of myeloid cells (Fig. 1E). Microglial repopulation, macrophage reduction, and lymphocyte infiltration were observed at 14 dpi (Fig. 1F). These results indicate immune cell heterogeneity in SCI.

### Infiltration of distinct dendritic cell subsets after SCI

We identified conventional DC1 (cDC1), cDC2, injury-associated cDC (Ia cDC), plasmacytoid DCs, and migratory DCs (mig DCs) based on previous studies (Fig. 1B) [38, 39]. The number of total DCs increased after SCI, while the ratios of the DC subtypes changed over time (Fig. 1G–H and Supplementary Fig. S4A). We discovered that over 90% of DCs in the sham group were migDCs (Ccr7^+^) located in the meninges, which indicated that migDCs in the spinal cord borders can migrate to draining lymph nodes (Fig. 1H–J) [38]. MigDCs were related to antigen presentation, positive regulation of hydrolase activity, and responses to tumor necrosis factor (TNF) (Supplementary Fig. S4B). A new DCs subset (cluster 20) was identified, which we called Ia cDCs (Fig. 1H–I). A GO analysis showed that these DCs were associated with antigen processing and presentation, wound healing involved in the inflammatory response, and oxidative phosphorylation (Supplementary Fig. S4C). cDC2 was the predominant DC subtype at 14 dpi (Fig. 1H–I).

### Dynamic changes in neutrophils subsets after SCI

Neutrophils have been identified as the first peripheral leukocytes recruited to injured spinal cord sites [40]. We detected that neutrophils were divided into four subsets (clusters 11–14). Ly6g was highly expressed in clusters 12–14 but lowly in cluster 11 (Fig. 1K). Cluster 11 was defined as over-inflammatory neutrophils, cluster 12 as mature neutrophils, cluster 13 as semi-mature neutrophils, and cluster 14 as immature neutrophils according to the DEGs and a previous study (Fig. 1K) [41].

The proportion of each neutrophil subtype was verified using flow cytometric analyses. Total neutrophils expanded at three dpi and reduced at 14 dpi (Fig. 1L). Only a few mature neutrophils were found in the healthy spinal cord (Fig. 1M, N and Supplementary Fig. S4D). Acute injury-associated neutrophils largely increased at three dpi, which is related to pro-inflammation, phagocytosis, glial cell activation, fibroblast proliferation, and axon regeneration (Fig. 1N and Supplementary Fig. S4E). Mature neutrophils were the predominant neutrophils subtype at 14 dpi, whereas immature and semi-mature neutrophils increased (Fig. 1M, N). The enriched GO terms showed that immature neutrophils were associated with proliferation, angiogenesis, and regeneration (Supplementary Fig. S4F).

### Infiltration of distinct lymphocytic subsets after SCI

**Fig. 2:**
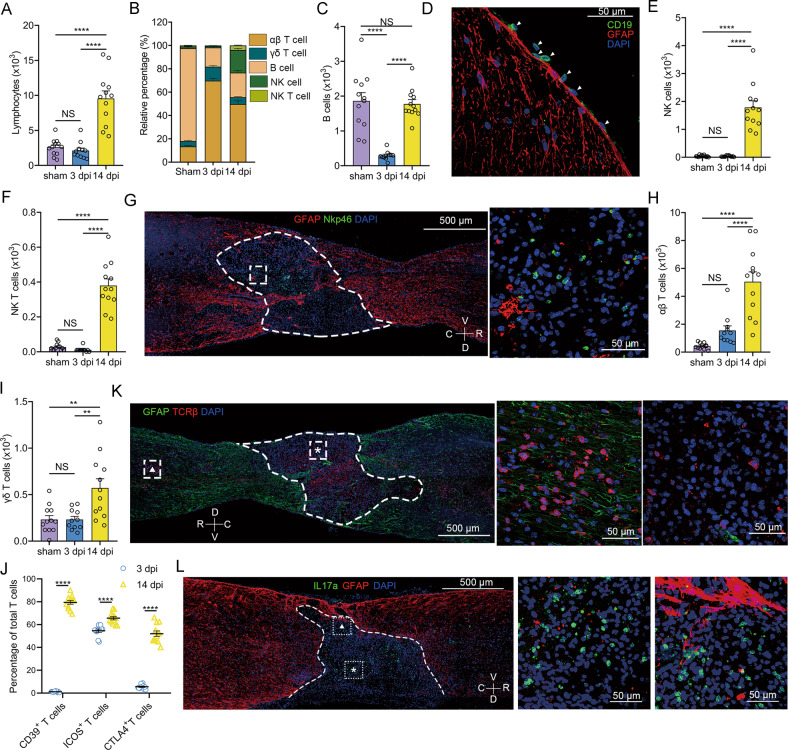
Lymphocyte heterogeneity after SCI. **A** Flow cytometric analyses show the absolute number of lymphocytes per mouse after SCI. One-way ANOVA with Tukey’s multiple comparisons posttest, *n* = 11 mice in the 3 dpi group, *n* = 12 mice in the sham and 14 dpi groups, ****P < 0.0001. **B** Flow cytometric analyses show the proportion of αβ T cells, γδ T cells, B cells, NK cells, and NK T cells in lymphocytes per mouse after SCI, respectively. **C** Flow cytometric analyses show the absolute number of B cells per mouse after SCI. One-way ANOVA with Tukey’s multiple comparisons posttest, ****P < 0.0001. **D** An IHC image of a transverse section in the sham group shows the location of B cells (white arrows). Images indicate similar results from three independent mice. Scale bar: 50 μM. **E**, **F** Flow cytometric analyses show the absolute number of NK cells **E** and NK T cells **F** per mouse after SCI, respectively. One-way ANOVA with Tukey’s multiple comparisons posttest, ****P < 0.0001. **G** IHC images of sagittal sections show the location of NK cells at 14 dpi. A dashed white line outlines the lesion border. An enlarged image of a dashed white boxed area is displayed on the right. Images indicate similar results from three independent mice. Scale bar: 500 μM or 50 μM. V, veutro; D, dorsal; R, rostral; C, caudal. **H**, **I** Flow cytometric analyses show the absolute number of αβ T cells **H** and γδ T cells **I** per mouse after SCI, respectively. One-way ANOVA with Tukey’s multiple comparisons posttest, ****p < 0.0001; **P = 0.0025 (sham vs. 14 dpi); **P = 0.0031 (3 dpi vs. 14 dpi). **J** Flow cytometric analyses show the percentage of activated T cells at 3 and 14 dpi per mouse. Multiple unpaired two-tailed Student’s *t*-test, ****P < 0.0001. **K** IHC images of sagittal sections showing the location of αβ cells at 14 dpi. A dashed white line outlines the lesion border. Magnified images of the boxed area are displayed in the middle (asterisk) and right (triangle). Images indicate similar results from three independent mice. **L** IHC images of sagittal sections show the location of Th17 at 14 dpi. A dashed white line outlines the lesion border. Enlarged images of the boxed area are displayed in the middle (asterisk) and right (triangle). Images indicate similar results from three independent mice.

Both scRNA-seq and flow cytometric analyses indicated that the lymphocytes mainly infiltrated at 14 dpi (Fig. 2A). B cells were the main subset of lymphocytes in the sham group, whereas αβ T cell were the main subset at 3 and 14 dpi. In addition, NK and NK T cells infiltrated at 14 dpi (Fig. 2B and Supplementary Fig. S5A). B cells are harmful for SCI recovery [42, 43]. We detected a small number of resident B cells in the healthy spinal cord (Fig. 2C). B cells were reduced at three dpi but returned to normal levels at 14 dpi (Fig. 2C). Immunohistochemical (IHC) staining for CD19 showed that these B cells were located in the meninges (Fig. 2D).

NK cells have been reported to be involved in the induction of neural death by secreting granzymes, perforin, and IFN-γ [44, 45]. However, it remains poorly characterized in SCI. Both scRNA-seq and flow cytometric analyses revealed that NK cells and NK T cells infiltrated only at 14 dpi (Fig. 2E, F and Supplementary Fig. S5A). IHC staining for Nkp46 revealed that these cells were restricted to the core of the injured site (Fig. 2G). GO analysis of DEGs in NK cells showed that NK cells were related to cell killing, production of granzyme B and cytokines, phagocytosis, and cytolysis (Supplementary Fig. S5B).

T cells are divided into αβ T cells and γδ T cells. αβ T cells infiltrated and expanded in SCI (Fig. 2H). αβ T cells occurring at 3 dpi (αβ T cell1) were completely different from those occurring at 14 dpi (αβ T cell2) (Supplementary Fig. S5C). Ifi27l2a, Isg15, Ifit3, Bst2, and Ltb were upregulated in αβ T cell2, which was enriched for T cell activation and cytokine production (Supplementary Fig. S5D, E). γδ T cells were mainly observed at 14 dpi (Fig. 2I). Activated T cells (ICOS^+^ T cells or CD39^+^ T cells) and exhausted T cells (CTLA4^+^ T cells) increased at 14 dpi (Fig. 2J). These T cells widely infiltrated the lesion core and rim (Fig. 2K). γδ T cells are almost all activated Th17 cells located at the core sites of SCI (Fig. 2L and Supplementary Fig. S5F). These results indicated that lymphocytes mainly infiltrated the lesion core restricted by the glial scar at 14 dpi.

### Dynamic changes in macrophages and microglia after SCI

**Fig. 3:**
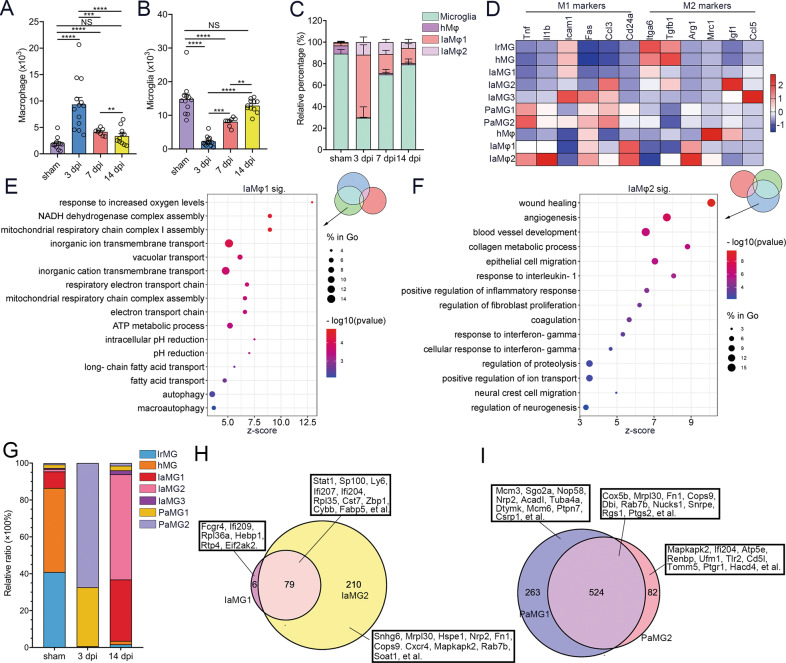
Microglia and macrophage heterogeneity after SCI. **A**, **B** Flow cytometric analyses show the absolute number of macrophages (**A**) and microglia (**B**) per mouse after SCI. One-way ANOVA with Tukey’s multiple comparisons posttest, *n* = 11 mice in the 14 dpi group, *n* = 12 mice in the sham and 3 groups, *n* = 12 mice in the 7 dpi group, **P < 0.01; ***P < 0.001; ****P < 0.0001. **C** Flow cytometric analyses show the proportion of different macrophage subtypes in total macrophage per mouse. hMφ, homeostatic macrophage; IaMφ, injury-associated macrophage. **D** A heat map shows the expression of M1 and M2 markers among all macrophage and microglia subsets. **E**, **F** Bubble diagrams of GO analysis (molecular function) of DEGs in cluster 9 (IaMφ1) (**E**) and 10 (IaMφ2) (**F**). **G** scRNA-seq reveals the proportion of different microglial subtypes in total microglia. hMG, homeostatic microglia; IrMG, interferon response microglia; IaMG, injury-associated microglia; PaMG, proliferation-associated microglia. **H**, **I** Venn diagrams show DEGs between IaMG1 and IaMG2 (**H**), PaMG1 and PaMG2 **I**.

We explored dynamic changes in macrophages and microglia after SCI. Peripheral macrophages largely infiltrated at 3 dpi but were reduced at 14 dpi (Fig. 3A). Resident microglia were reduced at 3 dpi and repopulated at 14 dpi (Fig. 3B). We identified three clusters of macrophage, including homeostatic macrophage (hMφ) and two clusters of injury-associated macrophage (IaMφ). IaMφ1 and IaMφ2 mainly infiltrated at three dpi (Fig. 3C and Supplementary Fig. S6A). The M1/M2 nomenclature was not applicable for the macrophage or microglia subtypes according to the expression of M1 markers (TNF, Il1b, Icam1, Fas, Ccl3, and Cd24a) and M2 markers (Itga6, Tgfb1, Arg1, Mrc1, Igf1, and Ccl5) (Fig. 3D). hMφ markedly overexpressed Ifi207, Ifi211, Cfh, Cyp27a1, Fam174a, CD48, Pea15a, and MHC II-associated genes (H2-Aa, H2-Ab1, CD74); IaMφ1 expressed Nrp2, Psmd1, Htr2b, Creg1, Uap1l1, Cenpb, Eif2s2, Ctsa, Slc6a8, and Renbp. IaMφ2 expressed Fn1, Dbi, Ptgs2, Tagln2, Capn2, Fam129b, Gsn, Zeb2, Rbms1, and CD44 (Supplementary Fig. S6B). Enriched GO term analyses indicated that IaMφ1 was related to increased oxygen levels, the respiratory chain, and autophagy, and IaMφ2 was related to wound healing and regeneration (Fig. 3E, F).

Microglia in our scRNA-seq data were divided into seven clusters, which contained interferon-response microglia (IrMG), homeostatic macrophages (hMG), and three clusters of injury-associated microglias (IaMG), and two clusters of proliferation-associated microglias (PaMG). There are two main clusters of microglia (IrMG and hMG) in healthy spinal cords. This distribution was sex-dependent. IrMG mostly expressed Eif2s3y (a male-specific gene), whereas hMG mostly expressed Xist (a female-specific gene) (Supplementary Fig. S6C).

IaMG1 and IaMG2 were primarily present at 14 dpi (Fig. 3G). There were 85 DEGs in IaMG1 and 289 DEGs in IaMG2 relative to hMG, indicating that IaMG1 was more similar to hMG than IaMG2. A Venn diagram demonstrated that IaMG1 significantly expressed Fcgr4, Ifi209, Rpl36a, Hebp1, Rtp4, and Eif2ak2, IaMG2 expressed Snhg6, Mrpl30, Hspe1, Nrp2, Fn1, Cops9, Cxcr4, Mapkapk2, Rab7b, Soat1, and there were 79 IaMG’s core genes including Stat1, Sp100, Ly6, Ifi207, Ifi204, Rpl35, Cst7, Zbp1, Cybb, and Fabp5 (Fig. 3H). IaMG core genes were enriched for GO terms associated with response to interferon, cytokine production, lipid metabolism, gliogenesis, glial cell migration, fibroblast proliferation, and antigen presentation (Supplementary Fig. S6D). Upregulated genes in IaMG2 were enriched for GO terms related to angiogenesis, neural crest cell migration, nerve development, and the semaphoring-plexin signaling pathway, indicating that IaMG2 may contribute to tissue repair (Supplementary Fig. S6E).

Two clusters of PaMG were the predominant microglial subtypes at three dpi (Fig. 3G). Analysis of DEGs compared to hMG showed 524 PaMG core genes (Cox5b, Mrpl30, Fn1, Cops9, Dbi, Rab7b, et al.), 263 PaMG1’s specific genes (Mcm3, Sgo2a, Nop58, Nrp2, Acadl, Tuba4a, et al.), and 82 PaMG2’s specific genes (Mapkapk2, Ifi204, Atp5e, Renbp, Ufm1, Tlr2, Cd5l, et al) (Fig. 3I). Enriched GO terms for the core genes showed that these PaMG genes were related to oxidative stress and proliferation (Supplementary Fig. S6F). PaMG1 was closer to a proliferative state, whereas PaMG2 was closer to the reaction to oxidative stress (Supplementary Fig. S6G, H).

### Microglia regress to a developmental growth state for repopulation after SCI

**Fig. 4:**
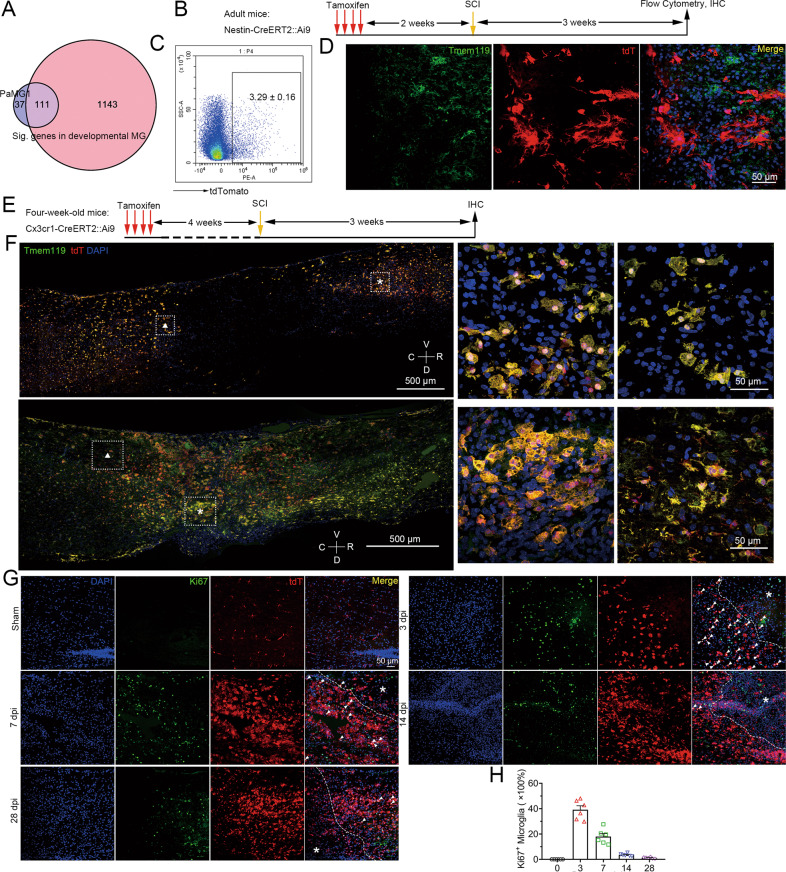
Microglia reverse to a development state for repopulation after SCI. **A** A Venn diagram shows DEGs in PaMG1 versus significant genes in developmental MG (published in Hammond et al., 2019, Immunity). **B** Experimental scheme for genetic lineage tracing of nestin^+^ cells after SCI. **C** Flow cytometric analyses show the proportion of microglia (CD45^low^Cx3cr1^+^) that express tdT at 21 dpi, *n* = three independent mice. **D** IHC images show no nestin^+^ cells co-express Tmem119, a microglial marker. Images indicate similar results from three independent mice. Scale bar: 50 μm. **E** Experimental scheme for genetic lineage tracing of Cx3cr1^+^ microglia after SCI. **F** IHC images show almost all microglia after SCI co-express tdT at 3 dpi (top panels) and 21 dpi (bottom panels). Magnified images of the boxed area are displayed in the middle (asterisk) and right (triangle). Images indicate similar results from three independent mice. Scale bar: 500 μm. Scale bar: 50 μm. **G** IHC images show the expression of Ki67 (a proliferative marker) in microglia after SCI at 0, 3, 7, 14, and 28 dpi. Asterisk: injured sites. Scale bar: 50 μm. **H** A statistic histogram of Ki67^+^ microglia. *N* = 6 independent mice per group.

Injured adult neurons have been reported to reverse the embryonic transcriptional state [46]. By scRNA-seq analysis, we were surprised to find 111 overlapping genes between PaMG1 in our study and developmental microglia as previously described, which accounted for 75% of the total DEGs (Fig. 4A) [9]. This indicates that early injured microglia are similar to developmental microglia, especially cluster 2b (a subtype of developmental microglia), as reported by Hammond et al., 2019, Immunity (Supplementary Fig. S7A) [9].

We next asked whether these PaMG were derived from nestin^+^ stem cells or dedifferentiated residual microglia. Nestin-CreER^T2^::Ai9 mice were established to track nestin-lineage cells. Two weeks after the administration of tamoxifen, SCI was induced in mice (Fig. 4B). After recovery for 28 days, only approximately 3.3% of microglia (CD45^low^CD11b^+^) positive for tdT were found, which indicated that most of the reborn microglia were not derived from nestin^+^ stem cells (Fig. 4C and Supplementary Fig. S7B). We did not find any cells co-expressing Tmem119 (a microglial marker) and tdT after SCI (Fig. 4D).

To determine whether residual microglia regressing to the developmental state is the reason for micriglial proliferation and replenishment, four-week-old Cx3cr1-CreERT2::Ai9 mice were administered tamoxifen for four days [47]. Four weeks after tamoxifen administration, only microglia that expressed tdT remained, whereas other Cx3cr1^+^tdT^+^ cells in the periphery were cleared (Fig. 4E and Supplementary Fig. S7C). We discovered that most microglia were co-labeled with tdTomato after SCI at 3 and 28 dpi, indicating that most reborn microglia originated from residual microglia (Fig. 4F). Microglia were highly proliferative after SCI, as revealed by the co-expression of Ki67 and tdTomato. Approximately 40% of microglia within the lesion rim expressed Ki67 at three dpi. The proportion of Ki67^+^ microglia decreased over time, with only approximately 0.5–5% of Ki67^+^ microglia were found at 14 and 28 dpi (Fig. 4G, H). These results indicated that residual microglia can regress to a developmental growth state for proliferation, which may account for microglial repopulation after SCI.

### Hif1α promotes microglial repopulation and functional recovery after SCI

**Fig. 5:**
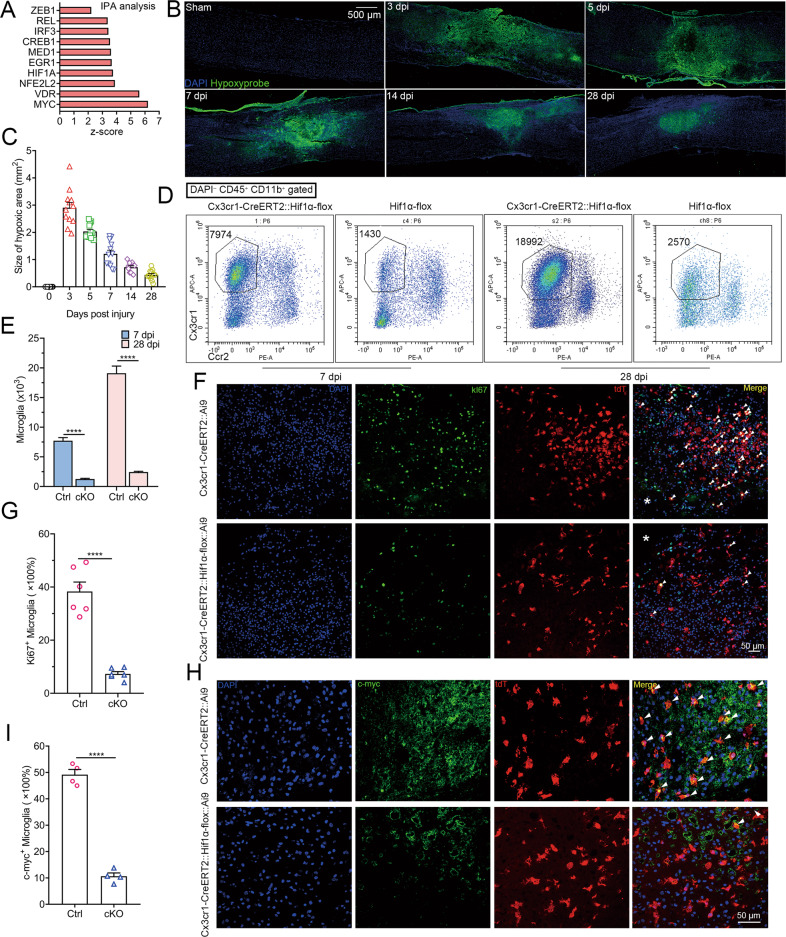
Hif1α promotes the proliferation and repopulation of microglia after SCI by maintaining their stemness. **A** Ingenuity Pathway Analysis showing the upstream regulatory factors for DEGs in PaMG1 compared to hMG. **B** IHC images reveal hypoxic conditions in the lesion sites (pimonidazole binding area) after SCI. **C** Statistical histogram of **B**, *n* = 12 sections from four independent mice per group. **D** Flow cytometric analyses showing the absolute number of microglia in Hif1α cKO mice and control mice after SCI at 7 and 28 dpi. Ccr2 was used to exclude monocyte-derived cells. **E** Statistical histogram of **D**. *n* = 6 independent mice per group, multiple unpaired two-tailed Student’s *t*-test, ****P < 0.0001. **F** IHC images showing proliferative microglia (Ki67^+^ tdT^+^ cells) at 3 dpi. Scale bar: 50 μm. **G** Statistical histogram for **F**. *n* = 6 independent mice per group, unpaired two-tailed Student’s *t*-test, ****P < 0.0001. **H** IHC images show the expression of c-myc in microglia (tdT^+^ cells) at 3 dpi. Scale bar: 50 μm. **I** Statistical histogram of **H**. *n* = 4 independent mice per group, unpaired two-tailed Student’s *t*-test, ****P < 0.0001.

To explore the regulatory factors for microglial repopulation, an upstream analysis of DEGs in PaMG1 was performed using IPA, which revealed that the Hif1α-Zeb1 axis may regulate microglia repopulation (Fig. 5A). Then, we explored the relationship between Hif1α and microglia proliferation. Intraperitoneal injection of pimonidazole was used to detect the hypoxic environment in the spinal cord after SCI. Pimonidazole was detected in these lesions (Fig. 5B). The size of the hypoxic area was the highest at three dpi and decreased over time, with a trend similar to the proliferation of microglia described above (Fig. 5C). These results indicated that microglial proliferation may be associated with hypoxia after SCI.

Cx3cr1-CreERT2::Hif1α-flox mice were used for Hif1α ablation in microglia. Hif1α is regarded as a key transcription factor for angiogenesis through the VEGF pathway [48]. IHC staining for CD31 (a specific marker of vascular endothelial cells) showed that Hif1α ablation in microglia partly inhibited revascularization (Supplementary Fig. S8A, B). The number of microglia was markedly decreased in Hif1α cKO mice compared to that in control mice at seven and 14 dpi, suggesting that Hif1α promotes microglial repopulation (Fig. 5D, E). We next explored whether this reduction was due to the diminished proliferation of microglia using Ki67 staining. The number of Ki67^+^ microglia was reduced by approximately 30% in Hif1α cKO mice at three dpi than in control mice (Fig. 5F, G). Since we demonstrated that microglia regress to a developmental growth state after SCI, we further assessed the stemness in microglia by the IHC staining of Nestin, c-myc, and Spp1 [49]. Nestin, c-myc, and Spp1 were highly expressed in microglia (approximately 38%, 50%, and 65%, respectively) at three dpi and were downregulated in Hif1α-deficient microglia (only approximately 6%, 10%, 12%, respectively) (Supplementary Figs. 5H, I, S8C–F). These results revealed that Hif1α expression in microglia after SCI promotes revascularization and microglial repopulation.

**Fig. 6:**
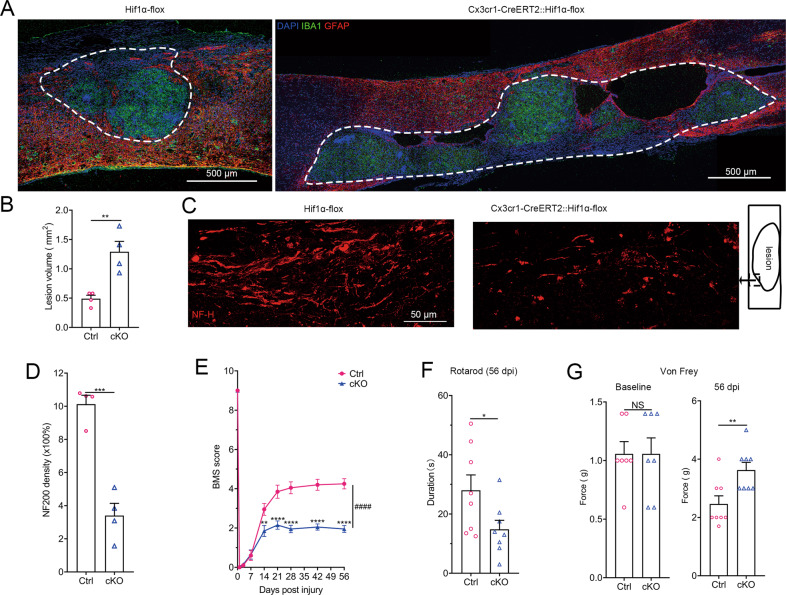
Hif1α is expressed in microglia and promotes functional recovery after SCI. **A** IHC images show the lesion sizes in Hif1α cKO and control mice at 56 dpi. The dashed white line indicates the lesion border. Scale bar: 500 μm. **B** Statistical histogram of **A**. *n* = 4 independent mice per group, unpaired two-tailed Student’s *t*-test, **P = 0.0052. **C** IHC images show axon fibers in the lesion (NF-H) in Hif1α cKO mice and control mice at 56 dpi. **D** Statistical histogram of **C**. *n* = 4 independent mice per group, unpaired two-tailed Student’s *t*-test, ***P = 0.0003. **E** BMS scores during the eight weeks of recovery after T10 contusion injury demonstrate an impaired functional recovery in Hif1α cKO mice compared with littermate controls with the same tamoxifen regimen. *n* = 10 mice per group, two-way ANOVA-RM with Bonferroni post hoc correction, F (1, 162) = 107.3 for the column factor, F (8, 162) = 333.1 for the row factor. **P = 0.0025, ****P < 0.0001, ^*####*^*P* < 0.0001. **F** Rotarod assays show impaired motor functional recovery in cKO mice compared to littermate controls at 56 dpi. *n* = 8 independent mice per group, unpaired two-tailed Student’s *t*-test, *P = 0.0459. **G** von Frey filament tests were performed at baseline and 56 dpi. *n* = 8 independent mice per group, unpaired two-tailed Student’s *t*-test, **P = 0.009.

We tested the effects of Hif1α ablation in microglia on functional recovery. Significantly larger lesion sizes were observed in Hif1α cKO mice than in control mice at 56 dpi (Fig. 6A, B). Tissue cavitation was observed in cKO mice. Immunostaining for neurofilament-H revealed that cKO mice displayed markedly fewer axon fibers than control mice (Fig. 6C, D). cKO mice exhibited worse recovery in hindpaw movement and placement, coordination, and trunk stability than littermate control mice according to the BMS scale (Fig. 6E). Hif1α cKO mice also displayed markedly worse motor functional recovery, as quantified by the rotarod test (Fig. 6F). Von Frey filament tests revealed sensory tactile deficits in cKO mice (Fig. 6G). Control and cKO mice showed similar baseline thresholds before SCI (Fig. 6G). Taken together, these results show that Hif1α expression in microglia is required for functional recovery.

## Discussion

We initially obtained a high-quality transcriptomic profile of immune cells in the injured microenvironment using scRNA-seq. Microglial reduction and infiltration of myeloid cells were present at three dpi, whereas microglial repopulation and of lymphocyte infiltration occurred at 14 dpi. Microglial repopulation is driven by the proliferation of residual microglia. Hif1α promotes microglial repopulation. Hif1α cKO mice displayed larger lesions, fewer axon fibers, and worse functional recovery than control mice.

We noted that NK cells were present at 14 dpi, which was related to cytotoxicity and inflammation. NK cells may worsen SCI. However, a recent study showed that gut microbiome-licensed meningeal IFNγ^+^ NK cells drive LAMP1^+^ TRAIL^+^ anti-inflammatory astrocytes [50]. Therefore, the role of NK cells in SCI should be further explored. Immature neutrophils have neuroprotective capabilities that drive axon regeneration by expressing NGF and IGF-1 [41]. In our study, a few immature neutrophils were newly discovered at 14 dpi. However, NGF and IGF-1 expression was not observed in these immature neutrophils (data not shown). Although GO enrichment analysis showed that these neutrophils were associated with regeneration, the detailed mechanisms need to be further explored.

The M1/M2 nomenclature was not applicable to the macrophage subtypes in SCI [51]. The expression of the M2 markers CD206 and Arg1 was different. CD206 was uniquely expressed in BAMs, whereas Arg1 was widely expressed in IaMφ. Almost all IaMφ were in the intermediate state, whereas IaMφ2 secreted more pro-inflammatory cytokines than IaMφ1. Post-phagocytosis M2 macrophages switch to the pro-inflammatory phenotype and have a poor capacity for phagocytosis [52]. We found that IaMφ1 was related to fatty acid transport, and autophagy, indicating that IaMφ1 is a type of phagocytizing macrophage. In contrast, IaMφ2 cells exhibited high inflammation and low phagocytic activity. IaMφ2 may be a type of post-phagocytic macrophage. IaMφ2 is also important for wound healing and angiogenesis. Thus, simply switching the macrophage phenotype from M1 to M2 for therapy is not sufficient.

The M1/M2 nomenclature was also not applicable in microglial subtypes. M1 markers (IL1β and TNF) and M2 markers (CD206 and Arg1) were widely expressed in PaMG, but were not expressed in hMG, IrMG, or IaMG. PaMG occurred mainly at three dpi in response to microglial reduction. We found that microglial repopulation occurred at 14 dpi. Microglial repopulation that forms a neuroprotective scar is important for recovery from SCI. Previous studies have shown that most repopulating microglia were derived from residual microglia after acute depletion or white matter injury [23, 53]. Our study found that most nestin^+^ stem cells could not differentiate into microglia after SCI. After permanently labeling microglia as tdT^+^ cells, reborn microglia also expressed tdT, which directly indicated that reborn microglia were mostly derived from residual microglia. Notably, microglia transiently express nestin at three dpi, which was similar to a previous study [23].

After validating the origin of the microglial repopulation in SCI, we considered how the residual microglia initiate proliferation. This study identified Hif1α as a key factor in microglial repopulation and functional recovery. Hif1α ablation in microglia causes larger lesion sizes and inflammatory diffusion, which may be due to a deficiency in wound compaction. Targeting Hif1α may help build a neuroprotective physical barrier for axon regeneration and functional recovery. It would be interesting to uncover whether similar results can be observed in humans after SCI. The functions of the activation of Hif1α in other cell types (astrocytes, neurons, oligodendrocytes, and fibroblasts) need to be examined.

## Supplementary information

Supplementary Figures

Fig. S1

Fig. S2

Fig. S3

Fig. S4

Fig. S5

Fig. S6

Fig. S7

Fig. S8

Fig. S9

Table S1

## Data Availability

The raw data files of ScRNA-seq have been deposited in the Gene Expression Omnibus with the following accession number: GSE182803. Additional data related to this paper can be requested from the corresponding author.
