# Supplementary material for: Single-cell transcriptome analysis reveals the immune heterogeneity and the repopulation of microglia by Hif1α in mice after spinal cord injury
Source: Cell Death Dis. 2022 May 3;13(5):432. doi: 10.1038/s41419-022-04864-z (PMC9065023; doi:10.1038/s41419-022-04864-z)
Supplement: Supplementary file 1 — Supplementary Figures [file 41419_2022_4864_MOESM1_ESM.docx]

**Supplementary Figure Legends**

**Figure S1.** ActD minimizes the artificially activation of immediate-early genes (IEGs) in process of single cell dissociation in vitro. A, Experimental design. Preparation of single-cell suspensions using a standard protocol or an ActD-based protocol for scRNA-seq. B, A t-SNE plot of 4,467 CD45^+^ cells isolated from the spinal cord with standard dissociation and ActD-based dissociation. hMG, homeostatic microglia; IrMG, interferon response microglia; DrMG, dissociation response microglia; BAM, border associated macrophage; MC, monocyte; MDC, monocyte-derived cell; Cbc, circulating basal cell. C, A t-SNE plot with the colors representing cells undergoing different dissociation. D, DEGs in the Standard-sham sample compared to the ActD-sham group.

E, The expression of 8 DEGs mentioned in (d) is analyzed by Real-time qPCR, n = 8 from 2 independent experiments, multiple unpaired two-tailed Student’s t-test, ****P* < 0.001. F, Violin plots show the expression of IEGs in each cluster.

**Figure S2.** Transcriptomic recognition of cell types in healthy spinal cords. A, Violin plots of transcriptional expression of markers of different immune cell types in each cluster. B, Proportion of cells in each cluster.

**Figure S3.** immune cell heterogeneity after SCI. A, Violin plots of the expression of markers of different immune cell types in each cluster after SCI. B, tSNE plots of CD45^+^ cells after SCI. C, Proportion of cells in each cluster. D, Violin plots of the expression of IEGs in each cluster.

**Figure S4.** DCs and neutrophils heterogeneity after SCI. A, scRNA-seq show the proportion of DC subtypes in total DCs after SCI. B and C, Bubble diagrams of GO analysis (molecular function) of DEGs in migDCs and Ia cDCs, respectively. D, scRNA-seq show the proportion of neutrophil subtypes in total neutrophils after SCI. E and F, Bubble diagrams of GO analysis (molecular function) of DEGs in Oi neutrophils and immature neutrophils, respectively.

**Figure S5.** Lymphocyte heterogeneity after SCI. A, scRNA-seq show the proportion of αβ T cells, γδ T cells, B cells, NK cells, and NK T cells in lymphocytes after SCI. B, A bubble diagrams of GO analysis (molecular function) of DEGs in NK cells. C, Proportion of αβ T cell1 and αβ T cell2 in each cluster. D, DEGs between in αβ T cell1 and αβ T cell2. E, A bubble diagram of GO analysis (molecular function) of DEGs in αβ T cell1. F, Violin plots of the expression of IFNg, Foxp3, IL17a, CD69, and ICOS in each cluster, respectively.

**Figure S6.** Macrophages and microglia heterogeneity after SCI. A, scRNA-seq show the proportion of macrophage subtypes in total macrophages after SCI. B, A Venn diagram shows DEGs among three macrophage subsets.tSNE maps of the expression of male (Eif2s3y) and female (Xist) markers. C, A tSNE maps of the expression of male (Eif2s3y) and female (Xist) markers. D and E, Bubble diagrams of GO analysis (molecular function) of core DEGs of IaMG (D) and IaMG signature genes (E). F-H, Bubble diagrams of analysis (molecular function) of core DEGs of IaMG (F), PaMG1 signature genes (G), and PaMG2 signature genes (H).

**Figure S7.** Microglia reverse to a development growth state after SCI. A, Venn diagrams show DEGs PaMG1 versus significant genes in each developmental microglial cluster. B, Gating strategy for microglia related to Fig. 4c. C, IHC images show only microglia express tdT in the spinal cord at the sham group. Magnified images of the boxed area are displayed in the middle (asterisk) and right (triangle). Images indicate similar results from three independent mice. Scale bar: 500 μm.

**Figure S8.** The expression of Hif1α in microglia promotes angiogenesis and maintains microglial stemness after SCI. A, IHC images show deficient angiogenesis in the lesion at 14 dpi in Hif1α cKO mice and control mice. A dashed white line outlines the lesion border. Scale bar: 500 μm. B, Statistical histogram of (A). n = 4 independent mice per group, unpaired two-tailed Student’s *t*-test, **P* = 0.0158*.* C, IHC images show the expression of Nestin in microglia within the lesion rim in Hif1α cKO mice and control mice at 3 dpi. Scale bar: 50 μm. D, Statistical histogram of (C). n = 4 independent mice per group, unpaired two-tailed Student’s *t*-test, *****P* < 0.0001*.* E, IHC images show the expression of Spp1 in microglia within the lesion rim in Hif1α cKO mice and control mice at 3 dpi. Scale bar: 50 μm. F, Statistical histogram of (E). n = 4 independent mice per group, unpaired two-tailed Student’s *t*-test, *****P* < 0.0001*.*

**Figure S9.** Gating strategy of analysis of various immune cells according to ScRNA-seq. A, Gating strategy of analysis of various lymphocytes subtypes related to Fig 2A-C, 2E, 2F, 2H, 2I. B, Gating strategy of analysis of activated T cells related to Fig. 2J. C, Gating strategy of analysis of various DC subtypes related to Fig. 1G-I. D, Gating strategy of analysis of various neutrophils subtypes related to Fig. 1L-N. E, Gating strategy of analysis of various macrophages subtypes and microglia related to Fig. 3A-C.
