# Supplementary figures and images for: Single-cell transcriptome analysis reveals the immune heterogeneity and the repopulation of microglia by Hif1α in mice after spinal cord injury
Source: Cell Death Dis. 2022 May 3;13(5):432. doi: 10.1038/s41419-022-04864-z (PMC9065023; doi:10.1038/s41419-022-04864-z)

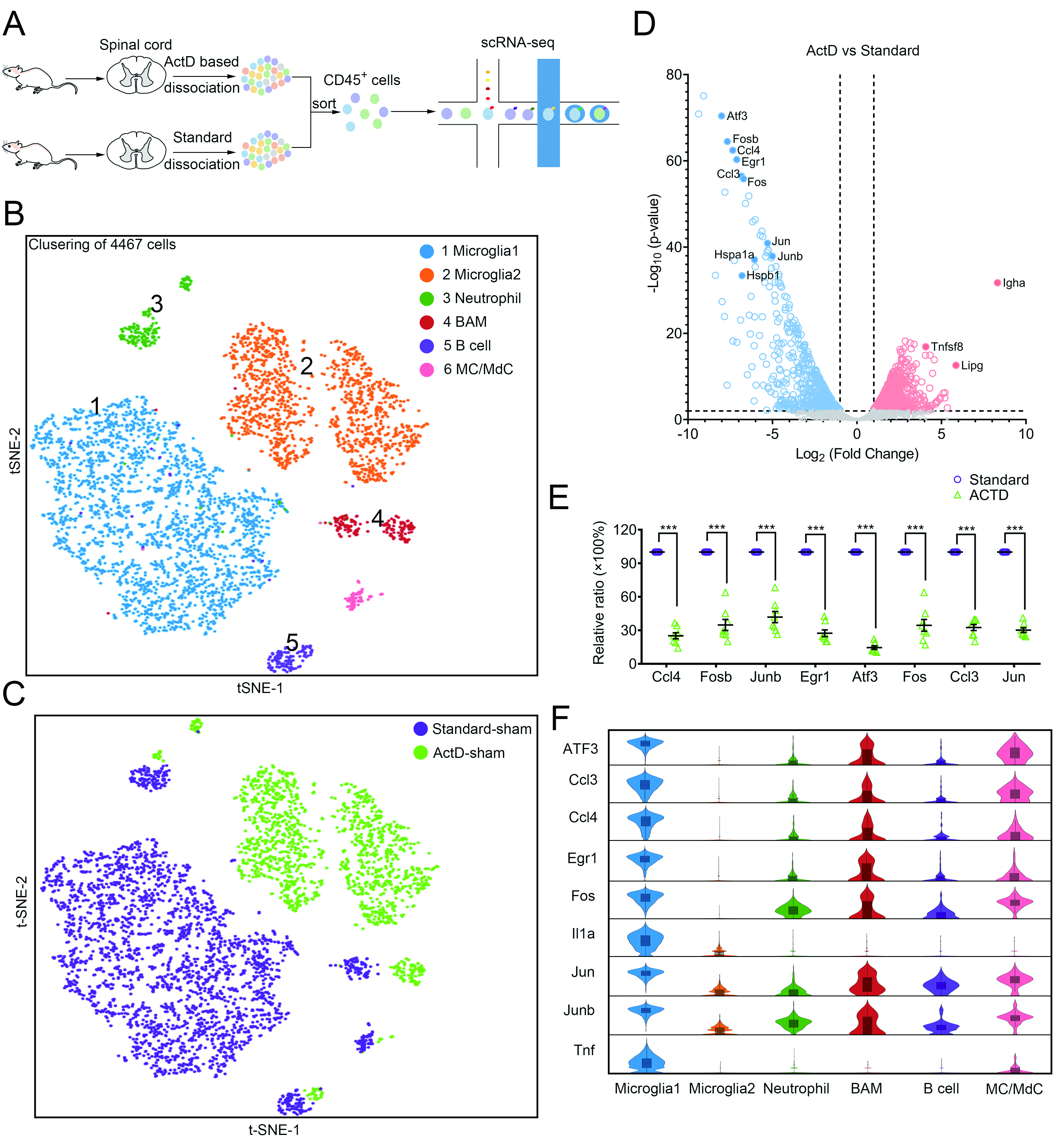

Supplement: Supplementary file 2 — Fig. S1 [file 41419_2022_4864_MOESM2_ESM.tif]

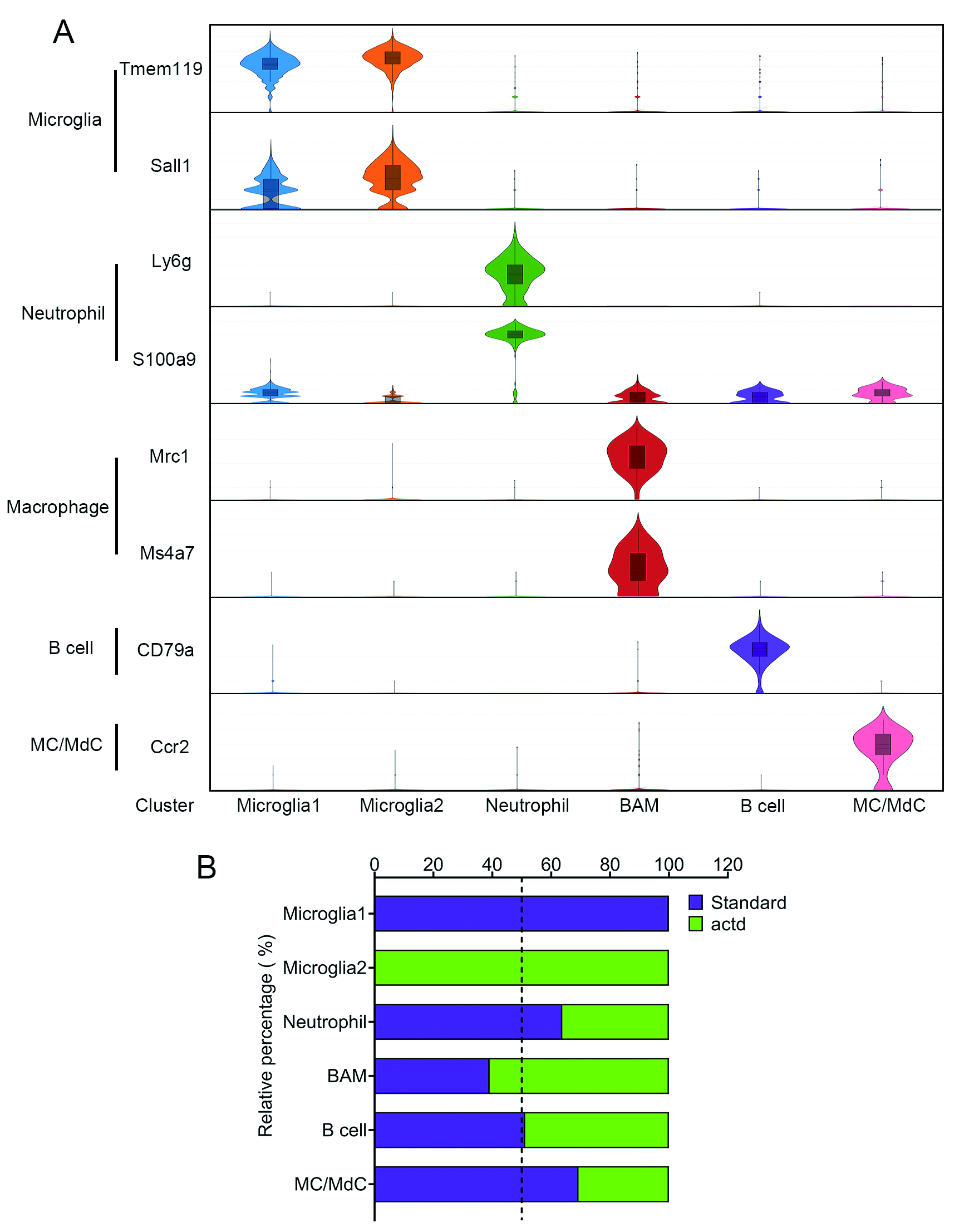

Supplement: Supplementary file 3 — Fig. S2 [file 41419_2022_4864_MOESM3_ESM.tif]

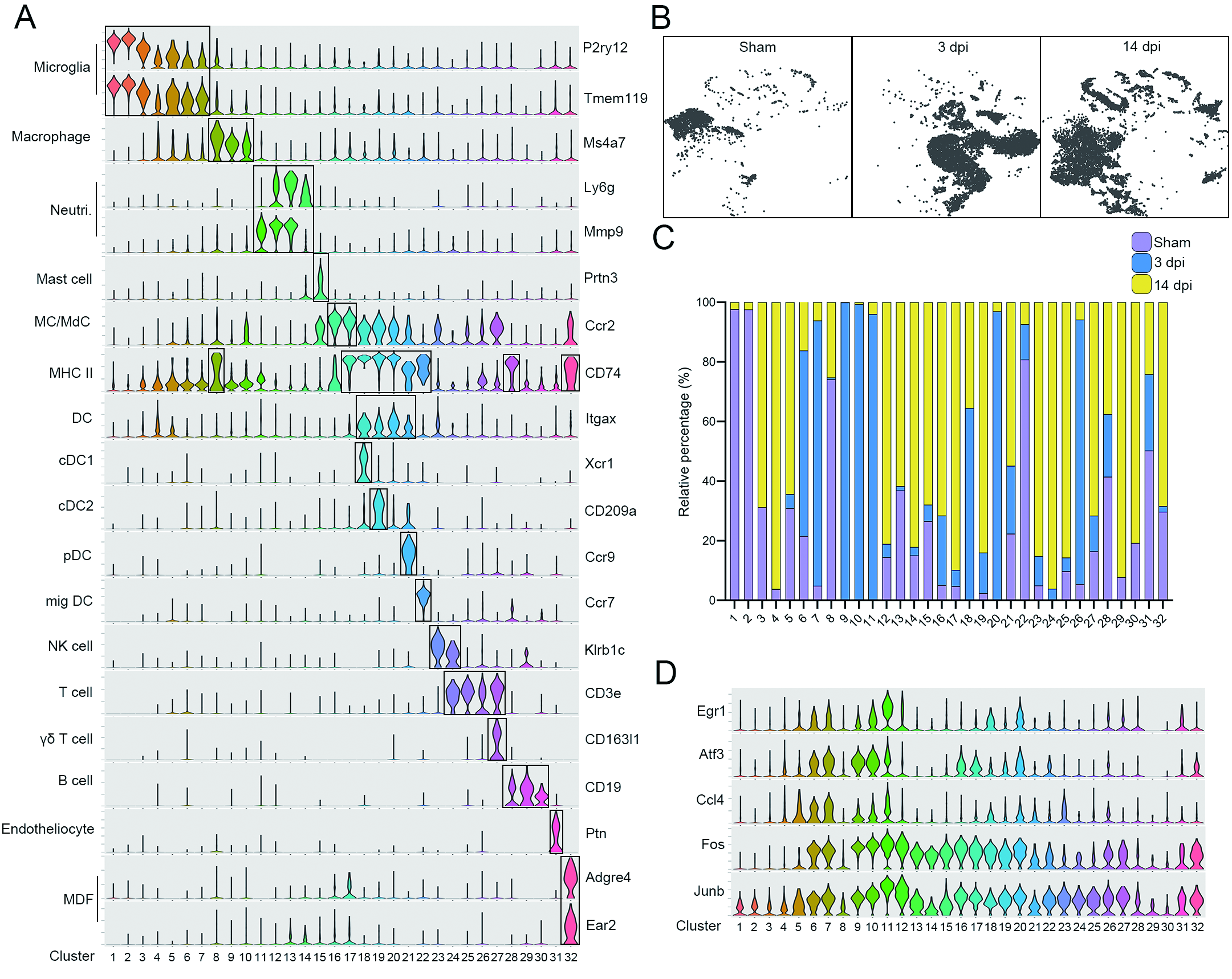

Supplement: Supplementary file 4 — Fig. S3 [file 41419_2022_4864_MOESM4_ESM.tif]

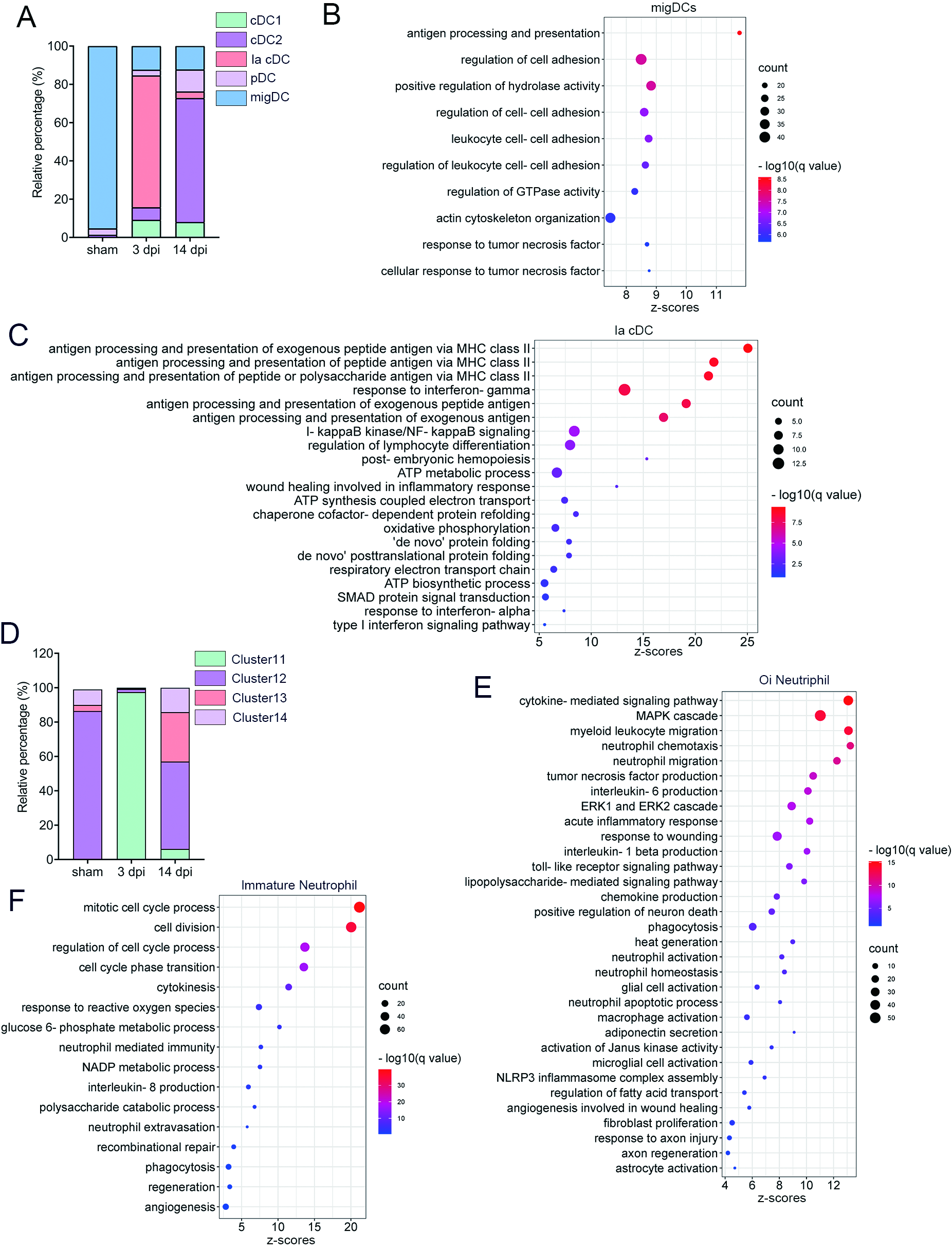

Supplement: Supplementary file 5 — Fig. S4 [file 41419_2022_4864_MOESM5_ESM.tif]

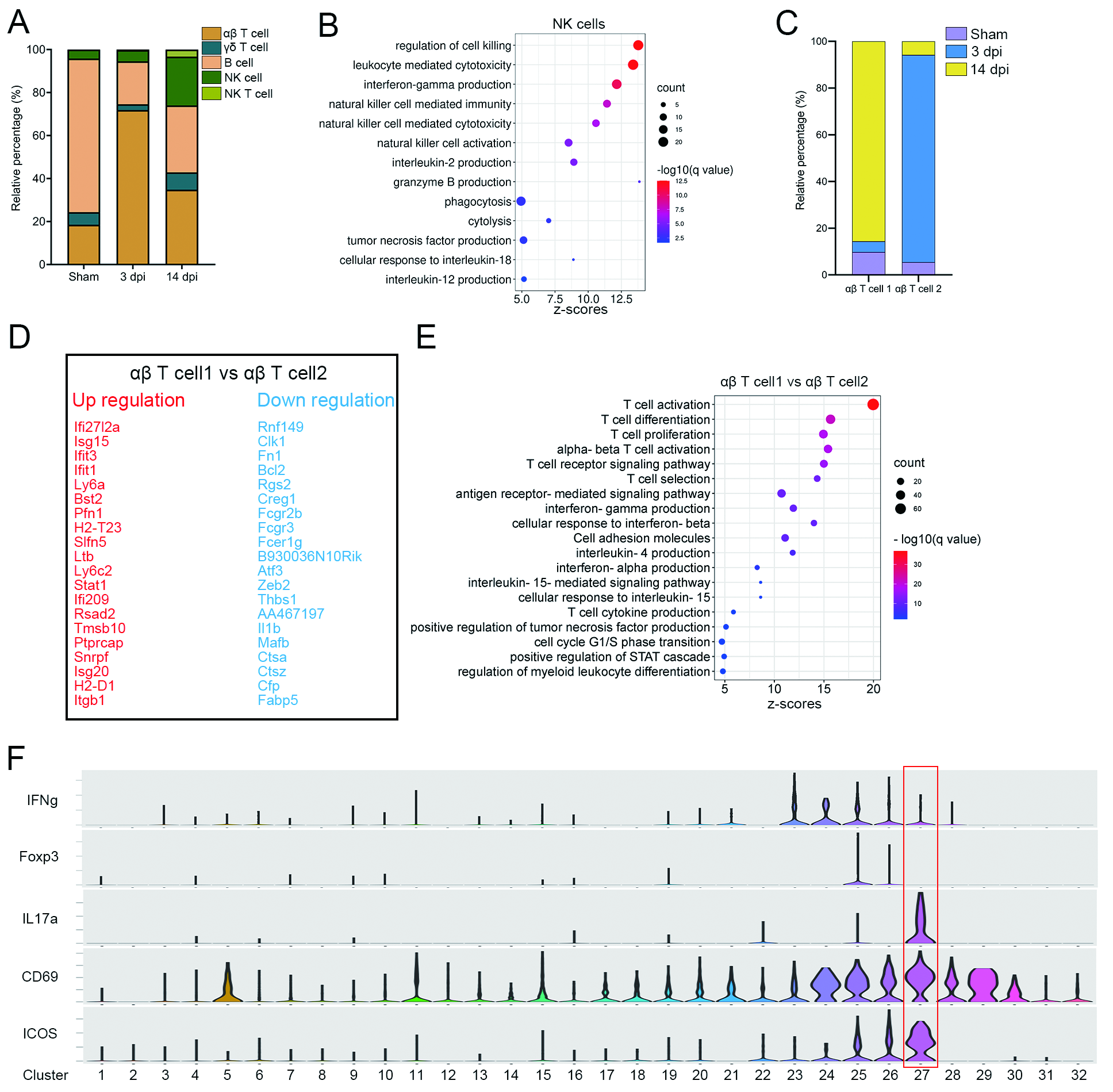

Supplement: Supplementary file 6 — Fig. S5 [file 41419_2022_4864_MOESM6_ESM.tif]

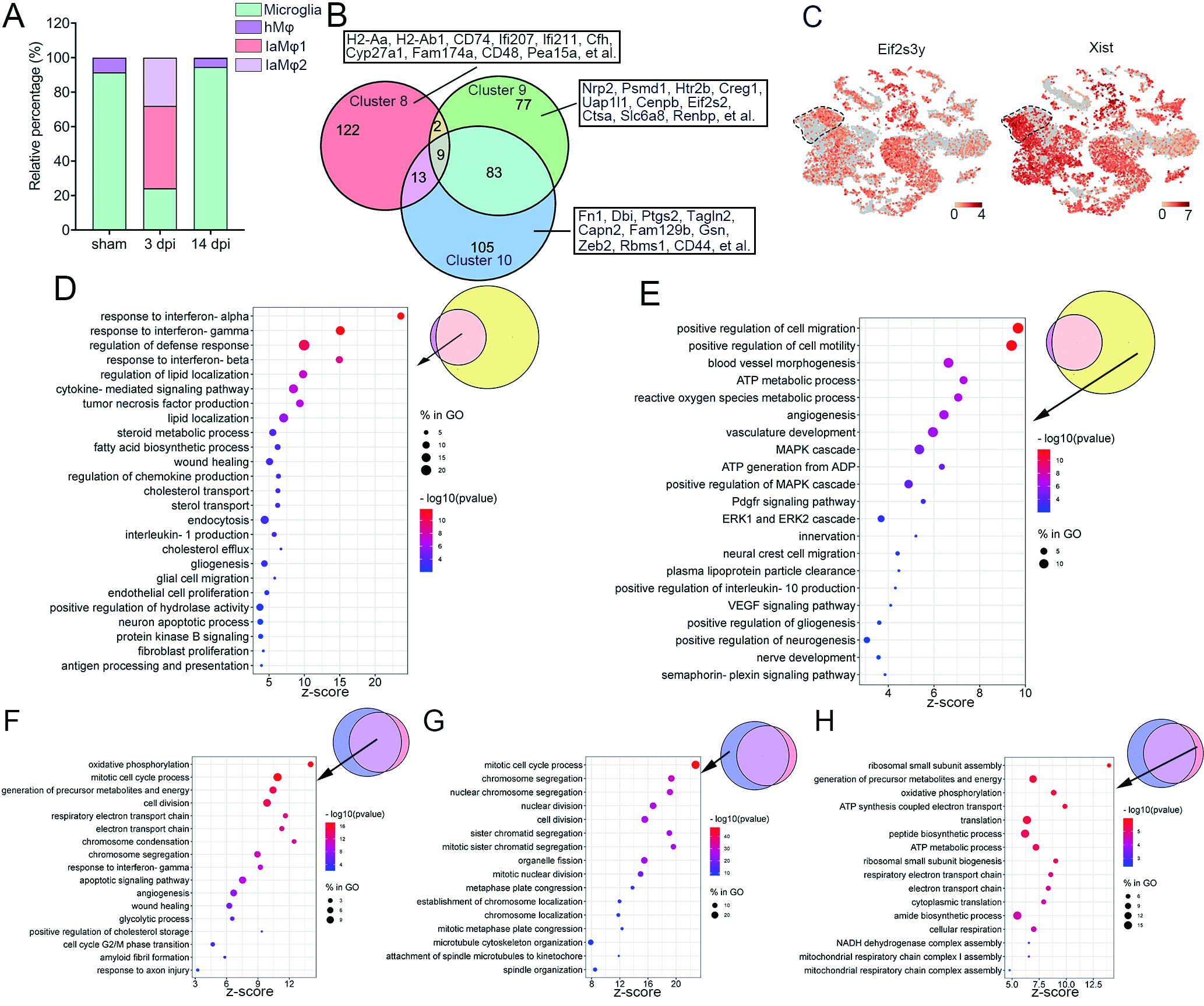

Supplement: Supplementary file 7 — Fig. S6 [file 41419_2022_4864_MOESM7_ESM.tif]

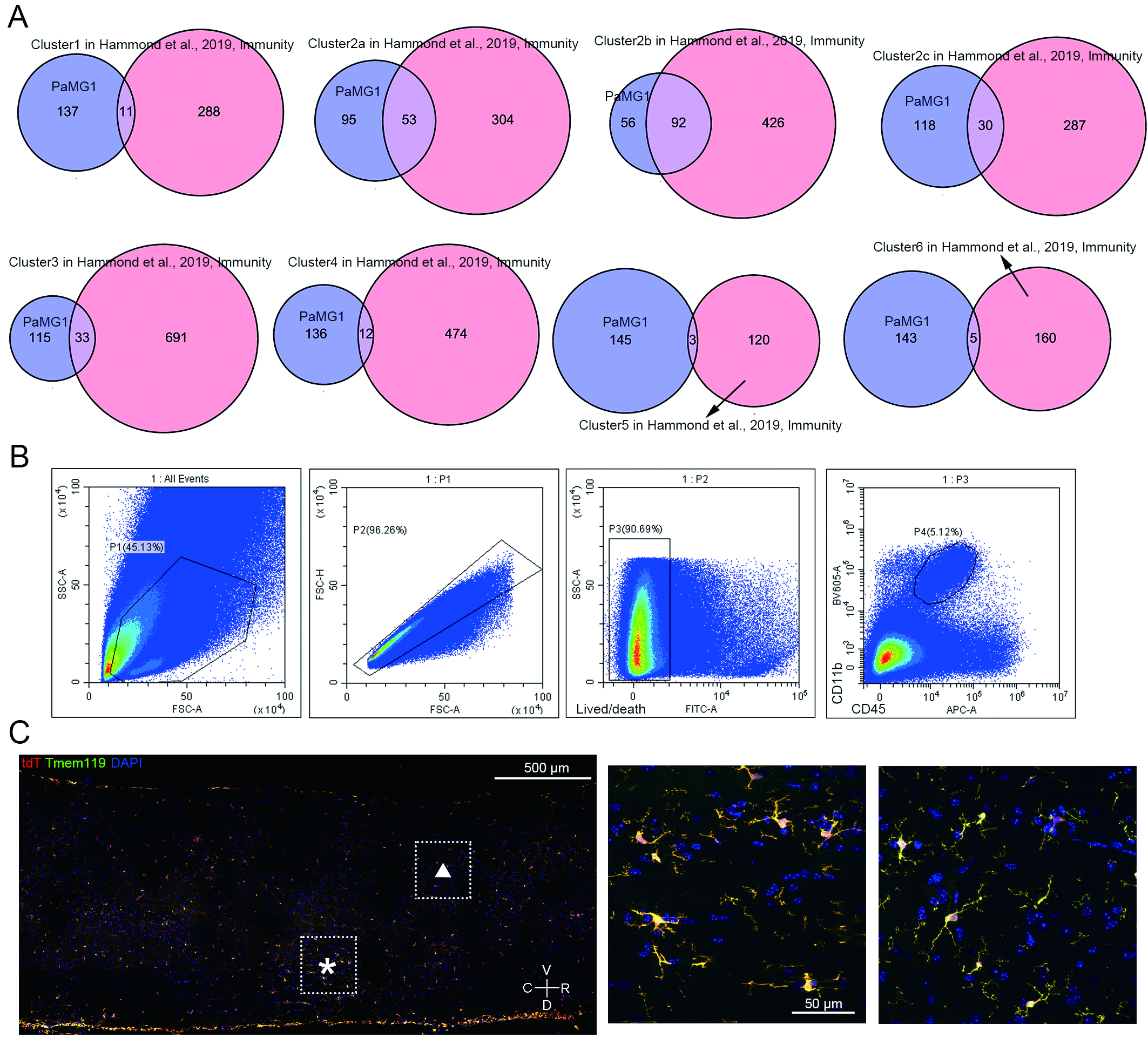

Supplement: Supplementary file 8 — Fig. S7 [file 41419_2022_4864_MOESM8_ESM.tif]

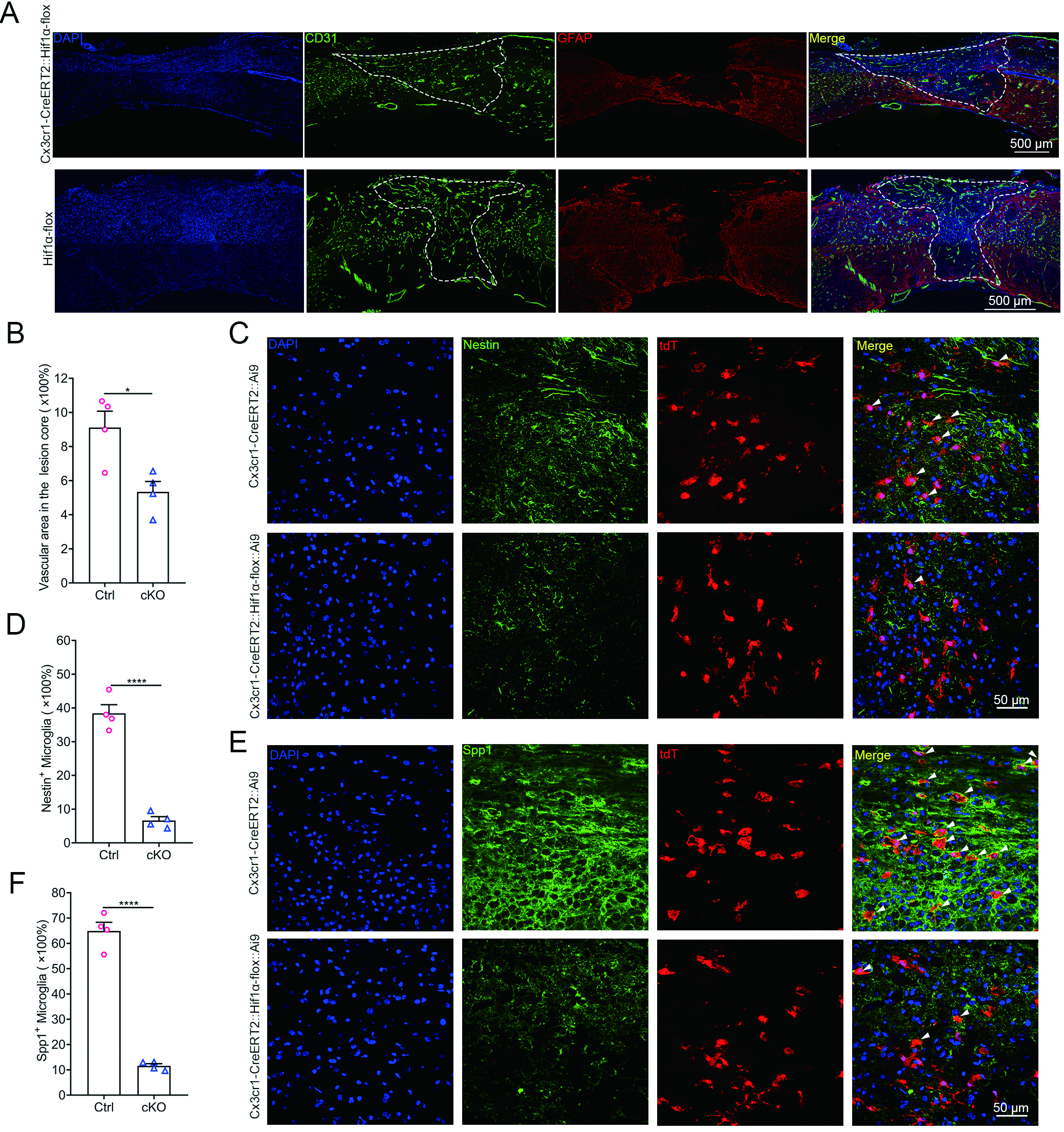

Supplement: Supplementary file 9 — Fig. S8 [file 41419_2022_4864_MOESM9_ESM.tif]

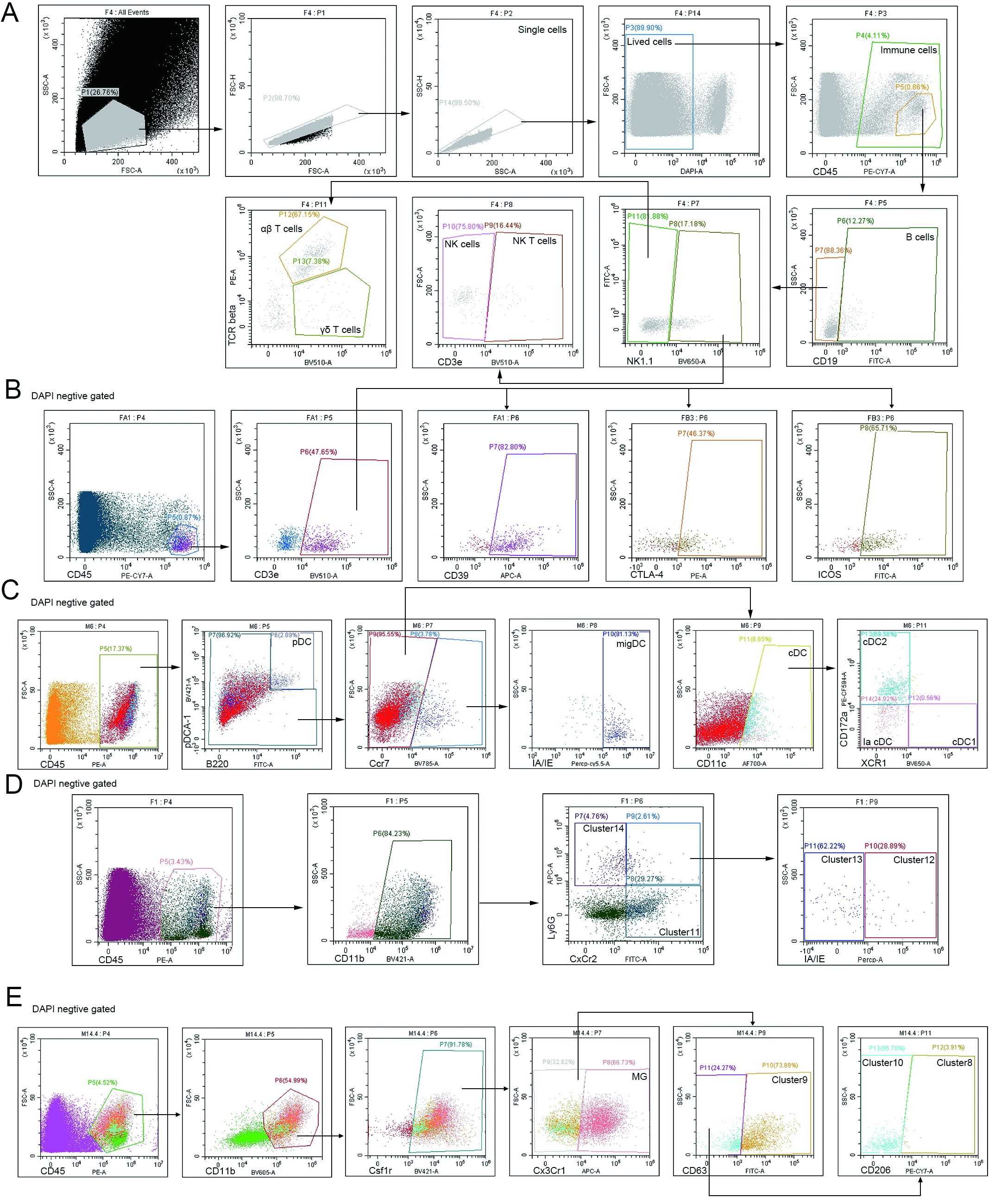

Supplement: Supplementary file 10 — Fig. S9 [file 41419_2022_4864_MOESM10_ESM.tif]
