# Supplementary material for: Single-cell transcriptome analysis reveals the immune heterogeneity and the repopulation of microglia by Hif1α in mice after spinal cord injury
Source: Cell Death Dis. 2022 May 3;13(5):432. doi: 10.1038/s41419-022-04864-z (PMC9065023; doi:10.1038/s41419-022-04864-z)
Supplement: Supplementary file 11 — Table S1 [file 41419_2022_4864_MOESM11_ESM.docx]

**TableS1. The details of materials**

| **REAGENT OR RESOURCES** | **SOURCE** | **IDENTIFIER** |
| --- | --- | --- |
| **Antibodies** | | |
| Rat anti-CD16/32 (clone 2.4G2) | BD Biosciences | Cat#553142;  RRID: AB_394657 |
| Rat anti-CD45, PE Conjugated (clone 30-F11) | BD Biosciences | Cat#553081;  RRID: AB_394611 |
| Hamster anti-TCR β chain, PE Conjugated (clone H57-597) | BD Biosciences | Cat#553172;  RRID: AB_394684 |
| Hamster anti-CD3, BV510 Conjugated (clone 145-2c11) | BD Biosciences | Cat#563024;  RRID: AB_2737959 |
| Mouse anti-NK1.1, BV650 Conjugated (clone PK136) | BD Biosciences | Cat#564143;  RRID: AB_2738617 |
| Rat anti-CD19, FITC Conjugated (clone 1D3) | BD Biosciences | Cat#557398;  RRID: AB_396681 |
| Rat anti-CTLA-4, PE Conjugated (clone UC10-4F10-11) | BD Biosciences | Cat#553720;  RRID: AB_395005 |
| Rat anti-ICOS, BB515 Conjugated (clone 7E.17G9) | BD Biosciences | Cat#564592;  RRID: AB_2738858 |
| Hamster anti-CD11c, AF700 Conjugated (clone HL3) | BD Biosciences | Cat#560583;  RRID: AB_1727421 |
| Rat anti-pDCA-1, BV421 Conjugated (clone 927) | BD Biosciences | Cat#566431;  RRID: AB_2739728 |
| Rat anti-CD11b, BV605 Conjugated (clone M1/70) | BD Biosciences | Cat#566431;  RRID: AB_2737951 |
| Human anti-CD39, APC Conjugated (clone REA870) | Miltenyi Biotec | Cat#130-114-358;  RRID: AB_2726584 |
| Rat anti-CD45, PE-cy7 Conjugated (clone 30-F11) | BioLegend | Cat#103114;  RRID: AB_312979 |
| Rat anti-Ccr7, BV785 Conjugated (clone 4B12) | BioLegend | Cat#120127;  RRID: AB_2716209 |
| Rat anti-IA/IE, PerCP-Cy5.5 Conjugated (clone M5/114.15.2) | BioLegend | Cat#107625;  RRID: AB_2191072 |
| Mouse anti-XCR1, BV650 Conjugated (clone ZET) | BioLegend | Cat#148220;  RRID: AB_2566410 |
| Rat anti-CD172a, PE/Dazzle 594 Conjugated (clone P84) | BioLegend | Cat#144015;  RRID: AB_2565279 |
| Rat anti-Cxcr2, FITC Conjugated (clone SA044G4) | BioLegend | Cat#149309;  RRID: AB_2566147 |
| Rat anti-Ly6G, APC Conjugated (clone 1A8) | BioLegend | Cat#127613;  RRID: AB_1877163 |
| Rat anti-CD11b, BV421 Conjugated (clone M1/70) | BioLegend | Cat#101235;  RRID:AB_10897942 |
| Rat anti-Csf1r, BV421 Conjugated (clone AFS98) | BioLegend | Cat#135513;  RRID: AB_2562667 |
| Mouse anti-Cx3Cr1, APC Conjugated (clone SA011F11) | BioLegend | Cat#149008;  RRID: AB_2564492 |
| Rat anti-CD63, FITC Conjugated (clone NVG-2) | BioLegend | Cat#143919;  RRID: AB_2876488 |
| Rat anti-CD206, PE-cy7 Conjugated (clone SA011F11) | BioLegend | Cat#141719;  RRID: AB_2562247 |
| Rat anti-CD45, APC Conjugated (clone 30-F11) | BioLegend | Cat#103112;  RRID: AB_312977 |
| Rat anti-CD19 | Thermo-Fisher | Cat#16-0193-81;  RRID: AB_657669 |
| Rat anti-IL17a, Alexa Fluor 488 Conjugated | Thermo-Fisher | Cat#53-7177-81;  RRID: AB_763579 |
| Rabbit anti-Ki67 | Thermo-Fisher | Cat# PA5-19462;  RRID:AB_10981523 |
| Chicken anti-GFAP | Abcam | Cat# ab4674;  RRID: AB_304558 |
| Rabbit anti-Ccr7 | Abcam | Cat# ab32527;  RRID: AB_726208 |
| Rabbit anti-Tmem119 | Abcam | Cat# ab209064;  RRID: AB_2800343 |
| Rabbit anti-IBA1 | Abcam | Cat# ab178846;  RRID: AB_2636859 |
| Rabbit anti-NF-H | Abcam | Cat# ab4680;  RRID: AB_304560 |
| Goat anti-Nkp | R and D Systems | Cat# AF2225;  RRID: AB_355192 |
| Goat anti-Spp1 | R and D Systems | Cat# AF808;  RRID: AB_2194992 |
| Goat anti-CD31 | R and D Systems | Cat# AF3628;  RRID: AB_2161028 |
| Mouse anti-c-Myc | Novus | Cat# NB600-302;  RRID: AB_2037060 |
| Chicken anti-Nestin | Novus | Cat# NB100-1604;  RRID: AB_2282642 |
| Chicken anti-RFP | Rockland | Cat#600-906-379;  RRID: AB_2614816 |
| Rabbit anti-RFP | Rockland | Cat#600-406-379;  RRID: AB_828390 |
| Alexa Fluor 488-AffiniPure F(ab')2 Fragment Donkey Anti-Rat IgG (H+L) | Jackson ImmunoResearch | Cat#712-546-153;  RRID: AB_2340686 |
| Alexa Fluor 594-AffiniPure Donkey Anti-Chicken IgY (IgG) (H+L) | Jackson ImmunoResearch | Cat#703-585-155;  RRID: AB_2340377 |
| Alexa Fluor 488-AffiniPure F(ab')2 Fragment Donkey Anti-Chicken IgY (IgG) (H+L) | Jackson ImmunoResearch | Cat#703-546-155;  RRID: AB_2340376 |
| Alexa Fluor 488-AffiniPure F(ab')2 Fragment Donkey Anti-Mouse IgG (H+L) | Jackson ImmunoResearch | Cat#715-546-150;  RRID: AB_2340849 |
| Alexa Fluor 488-AffiniPure F(ab')2 Fragment Donkey Anti-Rabbit IgG (H+L) | Jackson ImmunoResearch | Cat#711-546-152;  RRID: AB_2340619 |
| Alexa Fluor 488-AffiniPure F(ab')2 Fragment Donkey Anti-Goat IgG (H+L) | Jackson ImmunoResearch | Cat#705-546-147;  RRID: AB_2340430 |
| **Chemicals, peptides and recombinant proteins** | | |
| Hibernate-A medium | Thermo-Fisher | A1247501 |
| Percoll | GE Healthcare | 17089109-1 |
| Actinomycin D | Sigma-Aldrich | A1410 |
| DAPI | BD Biosciences | 564907 |
| **Critical commercial assays** | | |
| Neural Tissue Dissociation Kit (P) | Miltenyi Biotec | 130-092-628 |
| Hypoxyprobe kit | Hypoxyprobe, Inc | HP2-100KIT |
| **Deposited data** | | |
| SCI ScRNA-seq dataset | Current study | GSE182803 |
| **Experimental models: Organisms/strains** | | |
| Mouse: Hif1α^flox^ | SMOC | NM-CKO-190065 |
| Mouse: Ai9 | The Jackson Laboratory | JAX# 007909 |
| Mouse: Nestin^CreERT2^ | The Jackson Laboratory | JAX# 016261 |
| Mouse: Cx3Cr1^CreERT2^ | The Jackson Laboratory | JAX#021160 |
| **Software and algorithms** | | |
| imageJ | NIH | RRID: SCR_002798 |
| R studio |  | N/A |
| GraphPad prism 8 | GraphPad Software | RRID:SCR_002798 |
| FV31S-SW | Olympus | N/A |
| CyExpert | Cytoflex | N/A |
| Prism 8 | GraphPad | N/A |
| Metascape |  | http://metascape.org |
| Bioinformatics platform |  | http://www.bioinformatics.com.cn/ |
